# FUBP1 promotes neuroblastoma proliferation via enhancing glycolysis-a new possible marker of malignancy for neuroblastoma

**DOI:** 10.1186/s13046-019-1414-6

**Published:** 2019-09-11

**Authors:** Ping Jiang, Mao Huang, Weiwei Qi, Fenghua Wang, Tianyou Yang, Tianxiao Gao, Chuanghua Luo, Jing Deng, Zhonghan Yang, Ti Zhou, Yan Zou, Guoquan Gao, Xia Yang

**Affiliations:** 1 Program of Molecular Medicine, Affiliated Guangzhou Women and Children’s Medical Center, Zhongshan School of Medicine, Sun Yat-sen University, Guangzhou, China; 2 Department of Biochemistry, Zhongshan School of Medicine, Sun Yat-sen University, 74 Zhongshan 2nd Road, Guangzhou, 510080 China; 3 Department of Medical Oncology, Sun Yat-sen University Cancer Center, Guangzhou, China; 4 Guangdong Engineering & Technology Research Center for Gene Manipulation and Biomacromolecular Products, Sun Yat-sen University, Guangzhou, China; 5 Guangdong Province Key Laboratory of Brain Function and Disease, Zhongshan School of Medicine, Sun Yat-sen University, Guangzhou, China

**Keywords:** FUBP1, Neuroblastoma, Glycolysis, C-Myc, HIF1α, VHL

## Abstract

**Background:**

Neuroblastoma (NB) is one of the deadliest paediatric solid tumours due to its rapid proliferative characteristics. Amplified copies of MYCN are considered the most important marker for the prediction of tumour relapse and progression in NB, but they were only detected in 20–30% of NB patients, indicating there might be other oncogenes in the development of NB. The far upstream element binding protein 1 (FUBP1) was first identified as a transcriptional regulator of the proto-oncogene MYC. However, the expression and role of FUBP1 in NB have not been documented.

**Methods:**

FUBP1 expression was analysed from GEO database and verified by immunohistochemistry (IHC) and western blotting (WB) in NB tissues and cell lines. Cell proliferation and apoptosis were detected by Cell Counting Kit-8, Colony formation assay, EDU, TUNEL staining and flow cytometric analysis. Several glycolytic metabolites production was confirmed by ELISA and oxygen consuming rate (OCR). Luciferase assay, WB, chromatin immunoprecipitation (CHIP) were used to explore the mechanisms of the effect of FUBP1 on NB.

**Results:**

FUBP1 mRNA levels were increased along with the increase in International Neuroblastoma Staging System (INSS) stages. High expression of FUBP1 with low N-Myc expression accounted for 44.6% of NB patient samples (*n* = 65). In addition, FUBP1 protein levels were remarkably increased with NB malignancy in the NB tissue microarray (NB: n = 65; ganglioneuroblastoma: *n* = 31; ganglioneuroma: *n* = 27). Furthermore, FUBP1 expression was negatively correlated with patient survival rate but positively correlated with ki67 content. In vitro experiments showed that FUBP1 promotes NB cell proliferation and inhibits cell apoptosis via enhancing glycolysis and ATP production. Mechanistically, FUBP1 inhibited the degradation of HIF1α via downregulation of Von Hippel-Lindau (VHL), the E3 ligase for HIF1α, resulting in upregulation of lactate dehydrogenase isoform B (LDHB) expression to enhance glycolysis. Overexpressed or silenced N-Myc could not regulate FUBP1 or LDHB levels.

**Conclusions:**

Taken together, our findings demonstrate for the first time that elevated FUBP1 promotes NB glycolysis and growth by targeting HIF1α rather than N-Myc, suggesting that FUBP1 is a novel and powerful oncogene in the development of NB independent of N-Myc and may have potential in the diagnosis and treatment of NB.

## Background

Neuroblastoma (NB) originates from the nerve crest of the sympathetic nervous system and is one of the most common malignant extracranial solid tumours in children, accounting for approximately 10% of the malignant tumours in children and 15% of all paediatric cancer deaths [1]. According to the International Neuroblastoma Risk Group (INRG) classification system, NB can be divided into high-risk, intermediate-risk, low-risk, and extremely low-risk groups. Patients in very low-risk groups, such as partial M-S (INRG staging)/IV-S (INSS staging) patients, can self-resolve without any treatment [1, 2]. However, the progression-free survival rate of high-risk NB is low even with high-intensity comprehensive treatment, including chemotherapy, surgery, radiotherapy, autologous stem cell transplantation and immunotherapy [3]. NB has significant clinical and biological heterogeneity [4]. Therefore, there is no effective treatment for high-risk NB because the target of its pathogenesis is not clear.

MYCN, a member of the Myc family of transcription factors, is predominantly expressed in the developing neural crest and is a key regulator of fundamental cellular processes, including proliferation, differentiation and migration, with decreased levels associated with terminal differentiation [5]. The amplified MYCN copies are considered the most important marker for the prediction of tumour relapse and progression in NB [6]. The typical characteristic of MYCN amplification, which is consistently associated with drug resistance, advanced disease and poor outcome, is detected in 20–30% of all NB patients [7]. Although MYCN amplification is a proverbial marker of poor outcome, it cannot predict all poor survival outcomes [8]. Moreover, 70% of high-risk NB patients showed no amplification of MYCN [9], which indicated that other genetic alterations might play an important role in the development of NB.

The far upstream element binding protein 1 (FUBP1), also known as helicase V, is highly expressed in various tumour tissues and cell lines, including liver cancer, squamous cell carcinoma, renal cell carcinoma, breast cancer, prostate cancer, bladder cancer and non-small lung cancer [10–14]. FUBP1 can form a complex with the distal far upstream element (FUSE) site regulating gene expression [15], including c-Myc, p21, Usp29, etc. [16, 17], which displays a broad spectrum of activities, such as promoting proliferation, cell cycle, invasion and metastasis of tumour cells [18–20]. However, the expression and role of FUBP1 in neurogenic tumours has never been reported.

Aerobic glycolysis is considered the metabolic signature for cancer owing to its close association with tumour growth and progression [21]. An increase in aerobic glycolysis, which is the ability to switch from an oxidative metabolism to glycolysis and the production of lactate even when oxygen is plentiful (Warburg effect), is a key characteristic of solid tumours, including NB [22]. The oncoprotein c-Myc contributes to malignancy by various mechanisms, including accelerating tumour glycolysis, driving cellular proliferation, blocking differentiation, increasing cell migration and inducing angiogenesis [23]. In addition to the role of c-Myc in tumour glycolysis, another significant transcription factor, HIF1α, could regulate the expression of key glycolytic enzymes involved in the reaction to hypoxia, such as hexokinase 2 (HK2), aldolase A, pyruvate kinase M, lactate dehydrogenase isoform A (LDHA) and phosphoglycerate kinase 1 [24–28], while the detailed mechanism is still unclear. Notably, recent studies have described the effects of c-Myc and HIFs on carbon metabolism, protein synthesis, and proliferation as interactional [29–32]. Therefore, we propose that FUBP1 is involved in NB rapid proliferation via promoting glycolysis by targeting c-Myc or/and HIF1α.

In the current study, we investigated the role of FUBP1, another important oncogene in the development of NB independent of N-Myc, to determine the hypothesis that FUBP1 accelerated glycolysis, resulting in NB cell proliferation. These collective findings of this study may provide new targets for the diagnosis and treatment for NB.

## Material and methods

### Human samples

Eighteen frozen peripheral neuroblastic tumour (pNT) tissue samples [10 neuroblastoma (NB), 6 ganglioneuroblastoma (GNB), 2 ganglioneuroma (GN)] and a total of 123 pNT paraffin-embedded tissue samples (65 NB, 31 GNB and 27 GN) were collected from the Guangzhou Women and Children’s Medical Center. The collection was in strict agreement with the institutionally approved guidelines, and each participant gave written informed consent. The pNT patients were clinically classified according to INPC, the Shimada System. The clinical and biological characteristics of the patients are described in Additional file 8: Table S1.

### Cell lines and culture

The neuroblastoma cell lines SH-EP, SK-N-BE (2), SK-N-SH, SK-N-BE (2) C, SK-N-AS, and Kelly were from by Professor Bo, Li [33] from Sun Yat-sen University School of Medicine. MRC-5 and HeLs (Human embryonic Lung fibroblasts) cells were from Dr. Ruizhong, Zhang. Primary mouse neuron cells were isolated and cultured from fetal mouse hippocampus tissues as previously described [34]. NB cells were cultured in DMEM. Primary mouse neuron cells were cultured with Neurobasal (Gibco, USA) medium supplemented with B27 (1:50, Thermo Fisher, USA). All culture medium was supplemented with 10% FBS and 1:100 penicillin/streptomycin.

### shRNA, siRNA, plasmids, lentivirus and transfection

All lentiviral vectors contained the puromycin resistance gene. Vectors encoding FUBP1 shRNAs were purchased from Hanbio Biotechnology (Shanghai, China) Co., Ltd. FUBP1 siRNA, HIF1α and control siRNA were purchased from RiboBio (Guangzhou, China). Plasmids encoding FUBP1 were purchased from Obio Technology (Shanghai, China) Corp, Ltd.; the lentiviruses were packaged, and cells were transduced and subjected to puromycin selection as previously described [35]. According to the manufacturer’s instructions, transfections were performed at approximately 60% confluency using RNAiMAX or Lipofectamine 2000 (Invitrogen, USA). After 24 h, confirmation of interference was carried out using real-time quantitative PCR (RT-qPCR) and Western blotting.

### RNA isolation and RT-qPCR

Total RNA was extracted using TRIzol (Invitrogen, USA) according to the manufacturer’s protocol. First-strand cDNA synthesis was performed using 500 nanograms of total RNA, and the RT-qPCR analysis system was performed using iQ SYBR Green Supermix and the iCycler Real-time PCR Detection System (Bio-Rad, USA). Relative mRNA quantities were determined using the comparative cycle threshold (2^-∆∆Ct^) method. β-actin was used for normalization. Primer sets for SYBR Green analysis of human Fubp1, Hif1α Ldha, Ldhb, Vhl, Hk2, Pdk1(phosphoinositide dependent protein kinase-1), Pkm, G6pd (Glucose-6-phosphate dehydrogenase), Fbp1 (Fructose-1,6-bisphosphatase) and β-actin are as follows: Fubp1 forward: TCTTTCTCAGCCCTAACCCA; Fubp1 reverse: CTTGTCCAAGAGCCATCTCCAT; Hif1α forward: ACTTGGCAACCTTGGATTGG; Hif1α reverse: TGTGCAGTGCAATACCTTCCA; Ldha forward: GTTCCACTTAAGGCCCCTCC; Ldha reverse: AGATATCCACTTTGCCAGAGACA; Ldhb forward: CTGGTAGGTTTCGGCTCAGG; Ldhb reverse: CTCGCCACACTTGA; Vhl forward: GCGTTCCATCCTCTACCGAG; Vhl reverse: ATCGTGTCCCTGCATCTC; Hk2 forward: CATCCAGAGGAGAGGGGACT; Hk2 reverse: ACCGGTGTTGAGAAGCTCTG; Pdk1 forward: CATCCAGAGGAGAGGGGACT; Pdk1 reverse: ACCGGTGTTGAGAAGCTCTG; Pkm forward: GTCTGGGAGGAAAGTCGCTC; Pkm reverse: GCTGGGCCAATGGTACAGAT; G6pdh forward GCCGGAACCTGGATTCTGAT; G6pdh reverse: TGCCTGGGTGCCAGTTAAAA; Fbp1 forward: GCGTCTAAAGGTTTCCGCGA; Fbp1 reverse: ACCAGCAATGCCATAGAGGTG; β-actin forward: GCACTCTTCCAGCCTTCCTT; β-actin reverse: GTTGGCGTACAGGTCTTTGC.

### Western blotting

Tissues and whole-cell lysates were extracted for total protein using SDS buffer. Protein concentrations were determined using a BCA protein assay kit (KeyGEN BioTECH Laboratories, China) according to the manufacturer’s protocol. Equal amounts of protein were subjected to western blotting analysis. Blots were probed with antibodies against FUBP1 (ABE1330, Merck Millipore, USA), LDHB (ab85319, Abcam, USA), LDHA (ab101562, Abcam, USA), VHL (ab140989, Abcam, USA), HIF1α (14,179 s, CST, USA), HK2 (#2106 s, CST, USA), c-Myc (13,987 s, CST, USA), N-Myc (84,406, CST, USA) and β-actin (A5441, Sigma, USA). HRP-conjugated anti-rabbit (Santa-Cruz, USA) and anti-mouse (Santa-Cruz, USA) secondary antibodies were used. Proteins were determined using ECL Plus Reagent (Merck Millipore, USA).

### Immunohistochemistry

The 123 paraffin-embedded tissue samples collected were first stained with HE. After HE stained analysis, a representative tumour tissue region (4 mm × 4 mm) was selected from each sample to be perforated to prepare the tissue microarray (Shanghai Outdo Biotech Co., Ltd., China). Tissue microarrays were deparaffinized and dehydrated with graded alcohol. Then, the samples were prepared according to the protocol as described previously [35]. The arrangement of the tissue microarray is shown in Additional file 1: Fig. S1A.

### H-score

Staining results were analysed by a pathologist who was blinded to clinical data. Immunohistochemical scoring of proteins was based on a semiquantitative method according to the percentage of positive cells and intensity of staining. The scoring of percentage of positive cells was as follows: 0, no staining or staining in < 25% of the tumor cells; 1, staining in 25 to 50% of the cells; 2, staining in 51 to 75% of the cells; 3, staining in 76 to 100% of the cells. The scoring of intensity was as follows: 0, negative; 1, weak; 2, moderate; 3, strong. H-Score was calculated as the sum of the products of the intensity and the percentage of positive cells. Cellular proteins (H-Score ≥ 5) were considered as high expression while proteins (H-Score < 5) were considered as low expression.

### Hypoxic cell culture

Cells were incubated for 24 h in the BINDER hypoxic incubator (Germany) in 1% O_2_, 5% CO_2_ and 94% N_2_ in a humidified atmosphere at 37 °C. Cells were incubated in the incubator to maintain hypoxia. Confirmation of hypoxic conditions was performed using western blotting to detect expression of HIF1α.

### Flow cytometric analysis

NB cell staining with Annexin V and PI was carried out using an Annexin V-FITC/PI Apoptosis Detection kit (Merck, Germany). A total of 1 × 10^6^ cells were incubated at 37 °C for 30 min before centrifugation to collect the cell pellet, then resuspended in a Ca^2+^-enriched binding buffer and analysed using a Beckman Coulter flow cytometry. Data were calculated using CellQuest software.

### Colony formation assay

Cells transfected with FUBP1 siRNA or control siRNA or vector encoding FUBP1 or empty vector (EV) were plated in 6-well dishes (500 cell/dish) and then incubated for 2 weeks for colony formation. After 14 days, cell colonies were then fixed in 4% polyformaldehyde and stained with 0.1% crystal violet. All colonies were counted separately for each sample, and the relative colony numbers were calculated.

### Cell counting Kit-8 (CCK8)

Cell proliferation was measured via cell viability with a Cell Counting Kit-8 (Dojindo, Japan). Cells were seeded into 96-well plates and cultured for 24 h. Then, 100 μl CCK8 reagent was added to 96-well plates and incubated for 4 h. The absorbance (OD450 nm) was measured using a microplate reader (TECAN, Switzerland) and calculated.

### Glucose consumption assay

Cells were seeded in 6-well plates and cultured for 6 h, changed with fresh culture medium and incubated for additional 15 h. Glucose levels in culture medium were measured using a commercially Glucose Assay Kit (GAHK20, Sigma, USA). The absorbance (OD340 nm) of Glucose level was measured using a microplate reader (TECAN, Switzerland) and calculated.

### ATP colorimetric assay

Approximately 1 × 10^6^ cells were lysed in 100 μl ATP assay buffer and then deproteinized using the Deproteinization Sample Preparation Kit. Then, 50 μl cell lysates were added into a 96-well plate. For each well, a total of 50 μl Reaction Mix was prepared according to the manufacturer’s instructions (K354–100, BioVision, USA); then, the samples were incubated at room temperature for 30 min. The absorbance (OD570 nm) of ATP in the sample well was measured in a microplate reader (TECAN, Switzerland) and calculated.

### Lactate dehydrogenase (LDH) assay

A total of 1 × 10^6^ cells or homogenized tissues (100 mg) were harvested in 2–4 volumes of cold assay buffer and then centrifuged to collect the supernatant. A total of 50 μl of Reaction Mix was prepared according to the manufacturer’s instructions (ab102526, Abcam, USA) and then incubated at room temperature for 30 min. The absorbance (OD450 nm) of LDH activity was measured using a microplate reader (TECAN, Switzerland) and calculated.

### Lactic acid (LD) assay

Whole cell lysates or homogenized tissues were prepared in assay buffer (0.02 ml deionized water, 1 ml enzyme working solution, 0.2 ml substrate) according to the manufacturer’s instructions (Jiancheng, Nanjing, China) and then incubated at 37 °C for 10 min. The absorbance (OD530 nm) of LD was measured using a microplate reader (TECAN, Switzerland) and calculated.

### Oxygen consumption rate (OCR) assay

The measurement of oxygen consumption rate was carried out using Seahorse Bioscience XF-96 extracellular flux analyzer (Seahorse Bioscience). NB cells were seeded in XF 96-well plate (Seahorse Bioscience) with Seahorse XF medium supplemented with 10 mM glucose, 1 mM sodium pyruvate and 2 mM glutamine. The plate was pretreated in non-CO_2_ condition for 1 h and sequentially injected oligomycin (inhibition of ATP synthase), FCCP (uncoupling agent of oxygen consumption from ATP production), and rotenone and antimycin A (inhibitor of complex I and III in electron transport chain of mitochondria) into each well, and then proceed in XF analyzer for OCR measurement and analysis.

### Luciferase assay

The luciferase-encoding HIF1α-promoter or VHL-promoter and control sequences were purchased from Hanbio Biotechnology (Shanghai, China) Co., Ltd. Cells were plated in a 96-well plate, transfected with luciferase, and at 48 h after transfection, the culture medium was renewed. Luciferase activities were measured by a commercial kit (SpectraMax M5) using the Dual-Luciferase Reporter Assay System (Promega, USA).

### Statistical analysis

The variability of the data is presented as the SD (mean ± SD) and was assessed with Student’s *t* test between two groups. For multiple groups, significant differences were determined using one-way ANOVA. Kaplan-Meier curves were generated between protein expression and overall survival time. The log rank test was used for statistical comparisons between two groups. Statistical significance was defined at *p* < 0.05.

## Results

### FUBP1 increased with the degree of NB malignancy and was negatively correlated with the survival rate independent of N-Myc

**Fig. 1:**
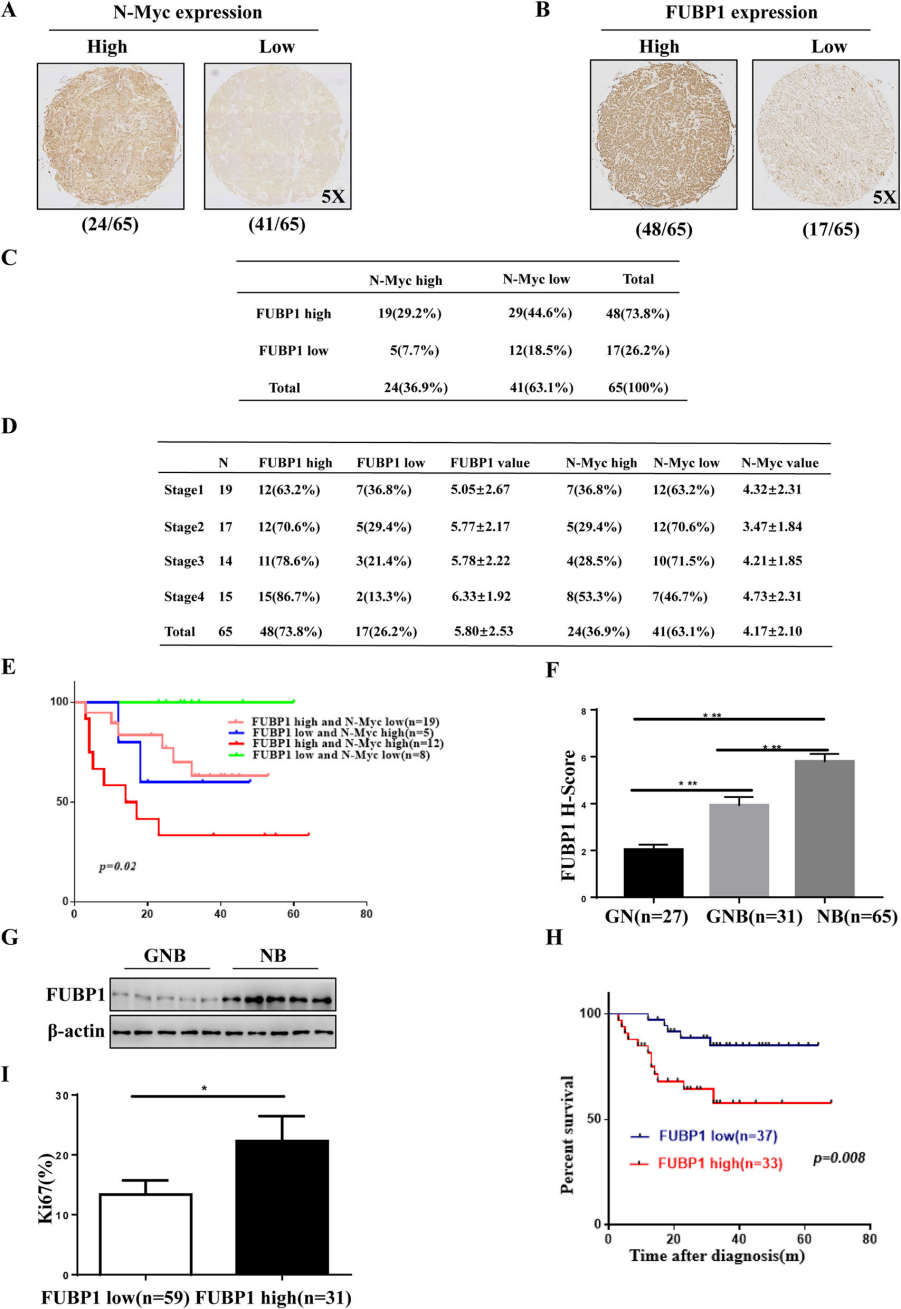
There was a significant positive correlation between FUBP1 and NB malignancy and a significant negative correlation with survival rate independent of N-Myc. (**a**) Immunohistochemistry staining of N-Myc and division into two categories: N-Myc high expression (*n* = 24) and N-Myc low expression (*n* = 41) according to the histochemistry score (H-score) of neuroblastoma TMA tissue (NB: *n* = 65). (**b**) Immunohistochemistry staining of FUBP1 and division into two categories: FUBP1 high expression (*n* = 48) and FUBP1 low expression (*n* = 17) according to the H-score on neuroblastoma TMA tissue (NB: n = 65). (**c**) Statistical analysis of the H-Score of FUBP1 and N-Myc in NB samples. (**d**) Statistical analysis of the H-Score of FUBP1 and N-Myc in the INSS NB stage 1–4.(**e**) Survival analysis of 44 NB pathologic subtype patients divided into four categories: high FUBP1 and low N-Myc expression (*n* = 19); low FUBP1 and high N-Myc expression (*n* = 5); high FUBP1 and high N-Myc expression (*n* = 12); low FUBP1 and low N-Myc expression (*n* = 8). High expression was considered a H-Score ≥ 5. (**f**) Statistical analysis of the H-Score of FUBP1 in NB TMA, which consists of 123 neuroblastoma tumour tissue samples (NB: *n* = 65; GNB: *n* = 31; GN: *n* = 27). (**g**) Western blot analysis of FUBP1 expression in frozen peripheral neuroblastic tumour (pNT) tissue samples (5 NB, 5 GNB). β-Actin served as a loading control. (**h**) Survival of NB patients with high expression of FUBP1 (H-Score ≥ 5; *n* = 33) versus those with low expression of FUBP1 (H-Score < 5; *n* = 37). (**i**) Statistical analysis of the ki67-positive ratio in high and low expression of FUBP1 in NB tissue from clinical data. Error bars represent the standard deviation (SD); one asterisk, *p* < 0.05; asterisks, *p* < 0.001

To verify the key role of N-Myc, we analysed N-Myc expression in an NB Tissue Microarray (TMA, Additional file 1: Fig. S1B). We found that the percentage of N-Myc high expression (H-Score ≥ 5; *n* = 24) in NB tissues (*n* = 65) was only 36.9%, while the percentage of N-Myc low expression (H-Score < 5; *n* = 41) in NB tissues (*n* = 65) was 63.1% (Fig. 1a and c). The data indicated that other genetic alterations might play an important role in the development of NB. To evaluate the relevance of potential genetic alterations in NB, we first analysed the NB transcriptome microarray from the GEO database and found that FUBP1 mRNA levels were increased with the progression of NB INSS stage (Additional file 1: Fig. S1D). Meanwhile, we found that the proportion of FUBP1 high expression was increased with the progression of NB INSS stage. Then, we tested FUBP1 levels in NB tissue microarrays of 123 neuroblastoma tumour tissue samples (NB: n = 65; GNB: *n* = 31; GN: *n* = 27, Additional file 1: Fig. S1C). Our data demonstrated that the proportion of the highly expressed FUBP1 group (H-Score ≥ 5; *n* = 48) was 73.8%, which was much higher than the proportion of the low expression FUBP1 group (H-Score < 5; *n* = 17, Fig. 1b-d). Interestingly, we found that the proportion of FUBP1 high expression and proportion of N-Myc low expression together accounted for 44.6% of NB pathological samples (n = 65) (Fig. 1c-d). Furthermore, we obtained 70 patients’ personal clinical follow-up information from patients with 44 NB pathological subtypes. Our data demonstrated that compared with simultaneous high FUBP1 expression and low N-Myc expression or high N-Myc expression and low FUBP1 expression, the survival outcomes of patients with both N-Myc and FUBP1 high expression were much poorer (*p* = 0.02; Fig. 1e). The data showed that FUBP1 might be another important oncogene in the development of NB independent of N-Myc. Next, we explored the key role of FUBP1 in the development of NB. Impressively, compared to GN tissue (H-Score = 2.04 ± 1.09), FUBP1 levels were remarkably increased in GNB tissue (H-Score = 3.94 ± 1.90; *p* < 0.001) and even more so in NB tissue (H-Score = 5.80 ± 2.53; *p* < 0.001; Fig. 1f and Additional file 1: Fig. S1C). FUBP1 levels were correspondingly significantly increased in NB compared to GNB tissue in 10 fresh tissues (NB: *n* = 5; GNB: n = 5, Fig.1g). Furthermore, we analysed the relationship between the survival time of 70 patients’ personal clinical follow-up information and FUBP1 expression. There was a significant negative correlation between FUBP1 and the survival rate of patients (*p* = 0.008; Fig. 1h). We found that the proportion of ki67-positive cells (ki67% = 13.84 ± 2.412; *n* = 59) in the low-expression FUBP1 group (H-Score < 5) was significantly lower than that in the high-expression FUBP1 group (H-Score ≥ 5; ki67% = 23.84 ± 3.86; *n* = 31; Fig. 1i) in all NB tissues, suggesting that FUBP1 may play an important role in the development of NB independent of N-Myc.

### FUBP1 promoted NB cell proliferation

**Fig. 2:**
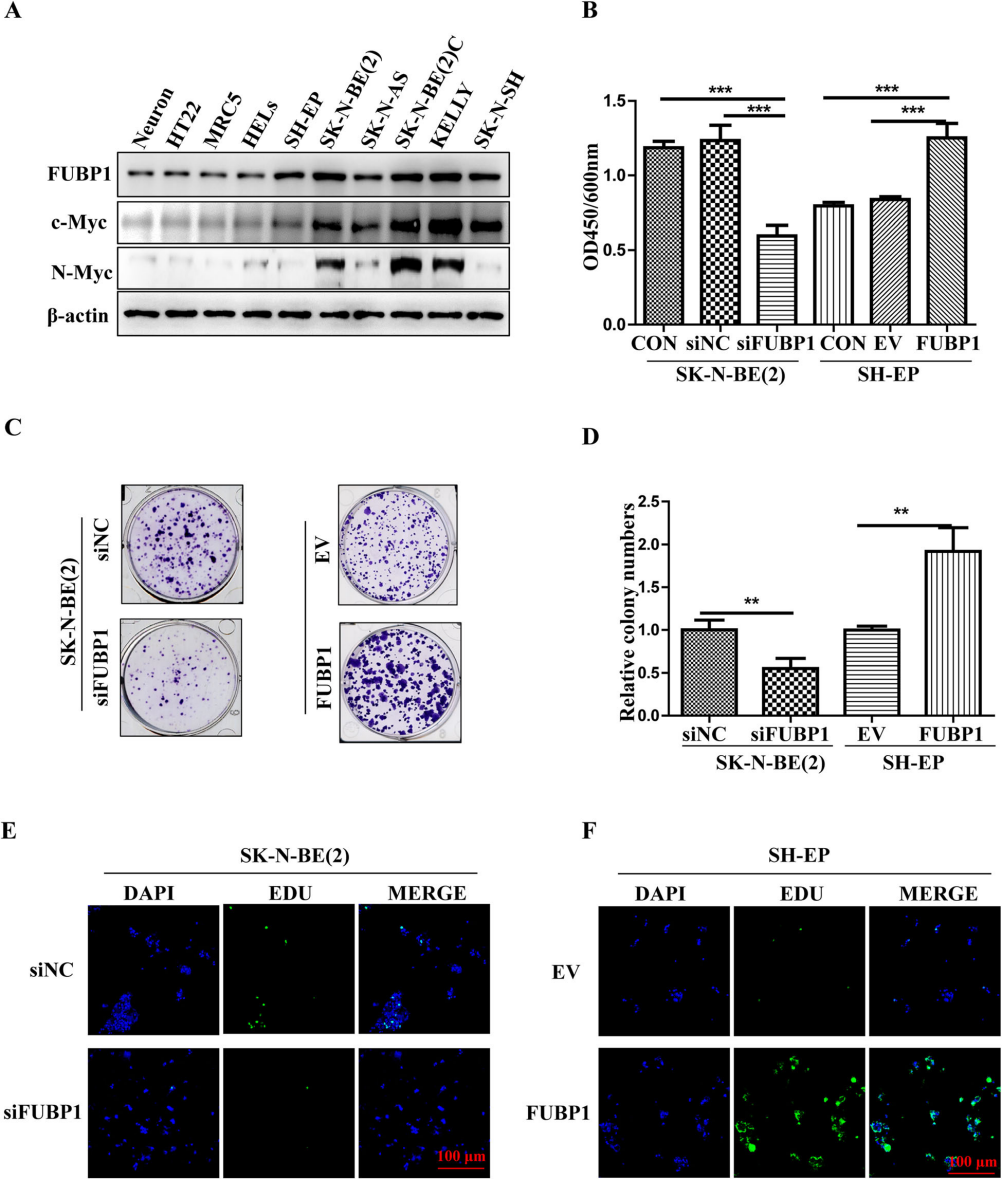
FUBP1 promotes NB cell proliferation. **a** Protein levels of FUBP1, c-Myc and N-Myc were determined by Western blot expression in NB cells and normal control cells. β-Actin served as a loading control. **b** SK-N-BE (2) cells were infected with siRNA to knock down FUBP1, and SH-EP cells were infected with viruses expressing FUBP1 for 72 h, followed by CCK8 analysis. EV: Empty vector; FUBP1: Vector with FUBP1 overexpression. **c** SK-N-BE (2) cells were infected with siRNA to knock down FUBP1 for 72 h, followed by a monoclonal plate formation assays. **d** Statistical analysis of relative colony numbers in each group. **e** SK-N-BE (2) cells were infected with siRNA to knock down FUBP1 for 72 h, followed by EDU staining. **f** SH-EP cells were infected with siRNA to knock down FUBP1 for 72 h, followed by EDU staining. Scale bar, 100 μm. Error bars represent the standard deviation (SD); two asterisks, *p* < 0.01; asterisks, *p* < 0.001

To determine the effect of FUBP1 on NB, we first tested FUBP1 levels in different NB cells [36]. Similar to the results in tissue, FUBP1 was significantly increased in the NB cell lines compared with normal cells (Neuron, HT22, MRC5, HELs; Fig. 2a). NB cells overexpressing or silencing FUBP1 (Additional file 1: Fig. S1F-H) were assessed by CCK8 and EDU analyses. The CCK8 results showed that the number of NB cells was significantly reduced after interference with FUBP1 (CON = 1.185 ± 0.44; siNC = 1.234 ± 0.10; siFUBP1 = 0.595 ± 0.07; *p* < 0.01), and the cell viability increased significantly after overexpression of FUBP1 (CON = 0.796 ± 0.02; EV = 0.839 ± 0.02; FUBP1 = 1.252 ± 0.10; *p* < 0.001; Fig. 2b). The interfering effects of siFUBP1 are shown in Additional file 1: Fig. S1G. Compared to the control, the number of single clones was significantly increased in NB cells after overexpression of FUBP1 and significantly reduced after interference with FUBP1 (Fig. 2c-d). EDU experiments showed that the proliferation of NB cells was significantly inhibited after interfering with FUBP1, while overexpression of FUBP1 significantly promoted NB cell proliferation (Fig. 2e and f). Taken together, these results indicated that FUBP1 could promote NB cell proliferation.

### FUBP1 inhibited apoptosis of NB cells

**Fig. 3:**
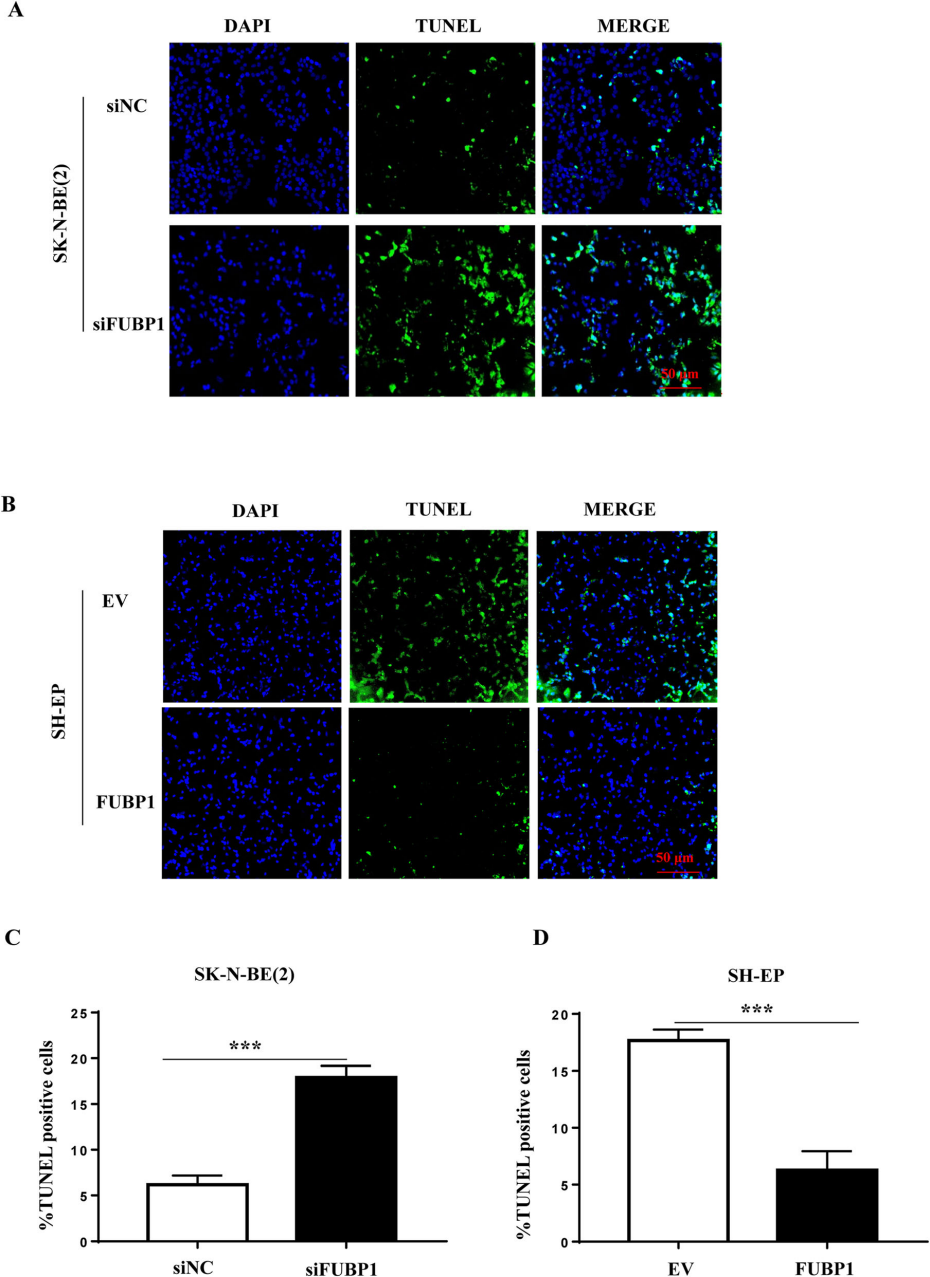
FUBP1 inhibits apoptosis of NB cells. (**a**) SK-N-BE (2) cells were infected with siRNA to knock down FUBP1 for 72 h, followed by TUNEL staining. (**b**) SH-EP cells were infected with viruses expressing FUBP1 for 72 h, followed by TUNEL staining. (**c**) Statistical analysis of the cell apoptotic rate after SK-N-BE (2) cells were infected with siRNA to knock down FUBP1 for 72 h. (**d**) Statistical analysis of the cell apoptotic rate after SH-EP cells were infected with viruses overexpressing FUBP1 for 72 h. Scale bar, 100 μm. Error bars represent the standard deviation (SD); one asterisk, *p* < 0.05

We further detected apoptosis of NB cells with overexpression or silencing of FUBP1. The TUNEL staining results showed that the staining intensity was increased after interference with FUBP1 and reduced after overexpression of FUBP1, indicating that FUBP1 inhibited NB cell apoptosis (Fig. 3a-d). Furthermore, NB cells were incubated with Annexin V and propidium iodide (PI) dye and detected by a flow cytometer. Our results showed that the apoptosis rate was significantly increased after interfering with FUBP1 (siNC = 7.32% ± 1.23; siFUBP1 = 13.88% ± 0.88; *p* < 0.05; Additional file 2: Fig. S2A and S2C), while high FUBP1 expression reduced the rate of apoptosis (EV = 15.90% ±1.82; FUBP1 = 7.13% ± 1.40; *p* < 0.05; Additional file 2: Fig. S2B and S2D). These results indicated that FUBP1 could inhibit NB cell apoptosis.

### FUBP1 promoted NB cell growth by enhancing NB glycolysis

**Fig. 4:**
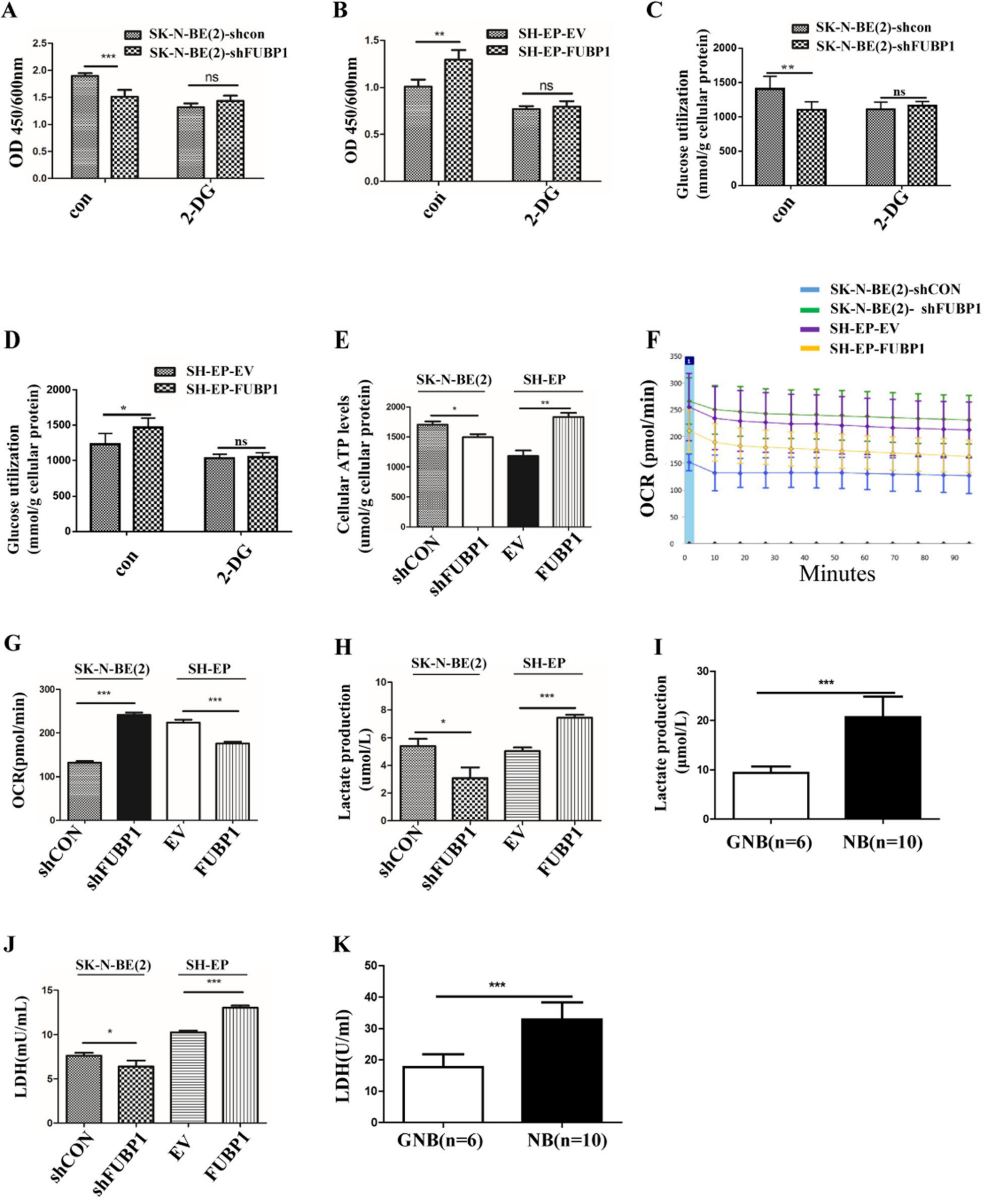
FUBP1 promotes NB cell growth through glycolysis. SK-N-BE (2) cells were stably infected with lentiviral containing shRNA specific to FUBP1 gene (shFUBP1) or shRNA containing scramble sequences (shCON) for 48 h. SH-EP cells were transfected with plasmid containing over-expressing FUBP1 gene sequences (FUBP1) or empty vectors (EV) for 48 h. The levels of FUBP1 levels in shFUBP1 or plasmid overexpressing FUBP1 cells were determined by Western blot. β-Actin served as a loading control. (**a**) SK-N-BE (2) cells were treated with 2-DG (2 mM) for 72 h, followed by CCK8 analysis. (**b**) SH-EP cells were treated with 2-DG (2 mM) for 72 h, followed by CCK8 analysis. (**c**) SK-N-BE (2) cells were treated with 2-DG (2 mM) for 72 h, followed by glucose consumption using a glucose assay kit. (**d**) SH-EP cells were treated with 2-DG (2 mM) for 72 h, followed by glucose consumption using a glucose assay kit. (**e**) Cell ATP production analysis. (**f**) Extracellular oxygen consumption analysis. (**g**) Statistical analysis of oxygen consumption analysis. (**h**) Lactate analysis in cell supernatant. (**i**) Lactate analysis in NB tissues (GNB: n = 6; NB: *n* = 10). (**j**) LDH activity analysis in cells. (**k**) LDH activity analysis in NB tissues (GNB: *n* = 6; NB: n = 10). Error bars represent the standard deviation (SD); one asterisk, *p* < 0.05; two asterisks, *p* < 0.01; asterisks, *p* < 0.001; NS means no significant difference

We detected the proliferation of NB cells that stably overexpressed or silenced FUBP1 by administration of the glycolytic inhibitor 2-DG (2 mM). The results showed that interference with FUBP1 inhibited the proliferation of NB cells (SK-N-BE(2)-EV = 1.90 ± 0.05; SK-N-BE(2)-shFUBP1 = 1.51 ± 0.13; *n* = 12; *p* < 0.001), while the additional administration of 2-DG could block the effect of FUBP1 on the proliferation of NB (SK-N-BE(2)-EV = 1.32 ± 0.07; SK-N-BE(2)-shFUBP1; n = 12; NS; Fig. 4a). Similarly, stable overexpression of FUBP1 promoted the proliferation of NB cells (SH-EP-EV = 0.94 ± 0.036; SH-EP-FUBP1 = 1.29 ± 0.10; n = 12; *p* < 0.01), and the additional administration of 2-DG blocked the effect of FUBP1 on the proliferation of NB (SH-EP-EV = 0.77 ± 0.03; SH-EP-FUBP1 = 0.80 ± 0.06; n = 12; NS; Fig. 4b). These results indicated that the effect of FUBP1 on NB proliferation was mainly through the glycolytic pathway.

To further identify whether FUBP1 could regulate NB glycolysis, the glucose utilization of NB was detected. The positive and negative results indicated that FUBP1 promoted glucose consumption by NB cells; the treatment of 2-DG blocked the effect of FUBP1 on the glucose consumption of NB cells (Fig. 4c-d). Furthermore, ATP content in NB cells after stable transfection with FUBP1 was detected. We found that ATP content in NB cells with stable interference of FUBP1 was significantly reduced (1607.00 ± 49.06 μmol/g) compared with the control (1706.00 ± 53.24 μmol/g; *p* < 0.05). Moreover, ATP content in NB cells with stable overexpression of FUBP1 was significantly increased (1834 ± 67.06 μmol/g; *p* < 0.01) compared with the control (1182 ± 91.05 μmol/g; Fig. 4e). We also examined the effect of FUBP1 on the oxygen consumption rate of NB cells. The results showed that the oxygen consumption rate increased (241.40 ± 5.47 pmol/min; *p* < 0.05) in the FUBP1 interference group compared with the control (132.40 ± 3.48 pmol/min). The rate of oxygen consumption was decreased (176.50 ± 4.13 pmol/min; *p* < 0.01) in the FUBP1-overexpressing group compared with the control (223.90 ± 6.56 pmol/min; Fig. 4f-g), suggesting that FUBP1 promoted the increase in ATP while reducing the oxygen consumption rate of NB cells. These results demonstrated that FUBP1 promoted the production of ATP by promoting aerobic glycolysis rather than the aerobic phosphorylation process in NB to provide energy for the rapid proliferation of NB.

Then, the content of lactic acid in NB cells was detected after interference with or overexpression of FUBP1. We found that lactate production was inhibited after interfering with FUBP1 (siNC = 5.40 ± 0.53 μmol/L; siFUBP1 = 3.08 ± 0.78 μmol/L; *p* < 0.05) but increased after overexpression of FUBP1 (EV = 5.03 ± 0.27 μmol/L; FUBP1 = 7.44 ± 0.21 μmol/L; *p* < 0.001; Fig. 4h). We found that lactic acid levels were much higher in NB tissues (20.70 ± 4.16 μmol/L; *n* = 10) than GNB tissues (9.40 ± 1.31 μmol/L; *n* = 6; *p* < 0.001; Fig.4i). In addition, compared to the control (7.61 ± 0.31 mU/ml), LDH activity was significantly decreased after interference with FUBP1 (6.40 ± 0.67 mU/ml; *p* < 0.05). LDH activity was significantly increased after overexpression of FUBP1 (EV = 10.24 ± 0.21 mU/ml; FUBP1 = 13.05 ± 0.23 mU/ml; *p* < 0.001; Fig. 4j). We also found that LDH activities were much higher in NB tissues (32.87 ± 5.47; n = 10) than GNB tissues (17.74 ± 4.07; n = 6; *p* < 0.001; Fig.4k), indicating that FUBP1 could promote LDH activity. Therefore, we concluded that FUBP1 could promote NB glycolysis.

### FUBP1 was positively correlated with the expression of LDHB

**Fig. 5:**
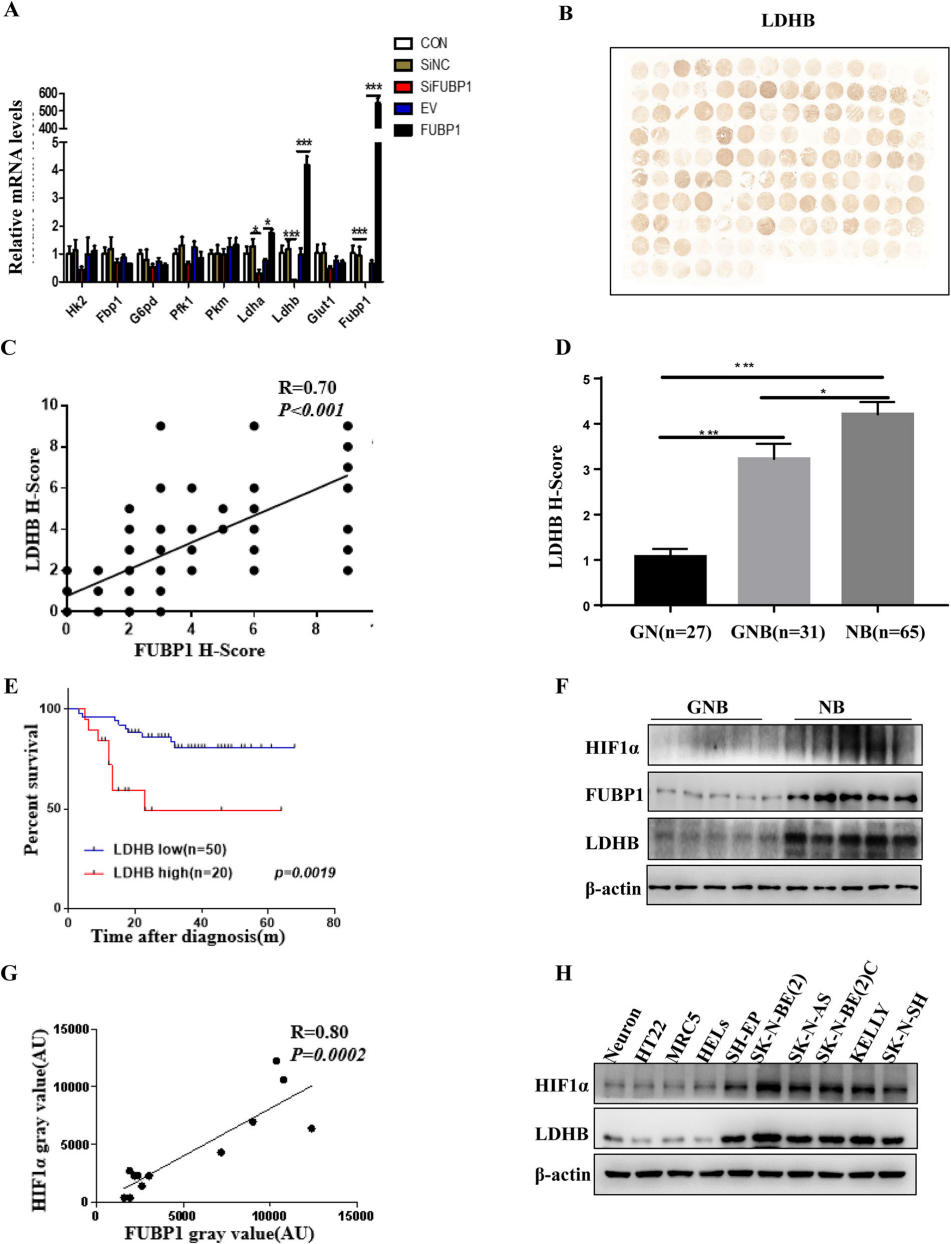
FUBP1 was positively correlated with the expression of HIF1α and LDHB. (**a**) qPCR analysis of glycolytic key enzymes genes expression, including: Hk2, G6pdh, Fbp1, Pfk1, Pkm, Ldha, Ldhb in the SH-EP cell, followed by transfecting with plasmid overexpressing FUBP1 or with siRNA to knock down FUBP1 was shown. (**b**) Immunohistochemistry staining of LDHB on NB TMA tissue. (**c**) Correlation analysis of LDHB and FUBP1 expression in NB TMA tissue. (**d**) Statistical analysis of the histochemistry score (H-Score) of LDHB in TMA. (**e**) Survival of NB patients with high expression of LDHB (H-Score ≥ 5) versus those with low expression of LDHB (H-Score < 5). (**f**) Western blot analysis of HIF1α and LDHB expression in frozen peripheral neuroblastic tumour (pNT) tissue samples (5 NB, 5 GNB, 2 GN). β-Actin served as a loading control. (**g**) The correlation analysis of FUBP1 and HIF1α expression in NB frozen tissues. (**h**) Western blot analysis of HIF1α and LDHB expression in NB cells and normal control cells. β-Actin served as a loading control. Error bars represent the standard deviation (SD); one asterisk, *p* < 0.05; asterisks, *p* < 0.001

To identify how FUBP1 regulates NB glycolysis, we first observed key glycolytic enzyme levels, including HK2, FBP1, G6PD, PKM, LDHA and LDHB, followed by FUBP1 overexpression or knockdown in NB cells. Figure 5a shows the expression of the key glycolytic enzyme mRNA levels, and we found that LDHA and LDHB mRNA levels were upregulated by FUBP1, while other enzymes obviously remained unchanged. Therefore, we hypothesized that LDHA and LDHB played a crucial role in the progression of NB. We first tested LDHA in the NB tissue microarray, our results showed that LDHA expression had no obvious correlation with survival ratio in those neuroblastoma samples (including 3GN, 23GNB, 44NB) (Additional file 3: Fig. S3A-C). Then we analysed the relationship between LDHA levels and survival outcome of NB patients (Pathological pattern is NB not GNB or GN, *n* = 44). Our data showed that LDHA expression was correlated with survival ratio (Additional file 3: Fig. S3D). Meanwhile, our data demonstrated that LDHA expression had the correlation with FUBP1 expression (Additional file 3: Fig. S3E). The results implied that LDHA could be regulated by FUBP1, but previous study demonstrated that LDHA promoted neuroblastoma growth independent of aerobic glycolysis [36]. Therefore, we focused on detecting LDHB expression in neuroblastoma.

Notably, the expression of LDHB in the NB tissue microarray was strongly positively correlated with FUBP1 (R = 0.7; *p* < 0.001; Fig. 5b and c). Furthermore, the histochemistry score of LDHB correspondingly significantly increased with the degree of NB malignancy in the clinical samples (Fig.5d). In addition, the survival ratio of patients with high LDHB expression was much lower than that in the low expression group (*p* < 0.01; Fig.5e). Thus, LDHB may be the downstream molecule of FUBP1 in the progression of NB.

### FUBP1 regulated downstream LDHB expression via targeting HIF1α

**Fig. 6:**
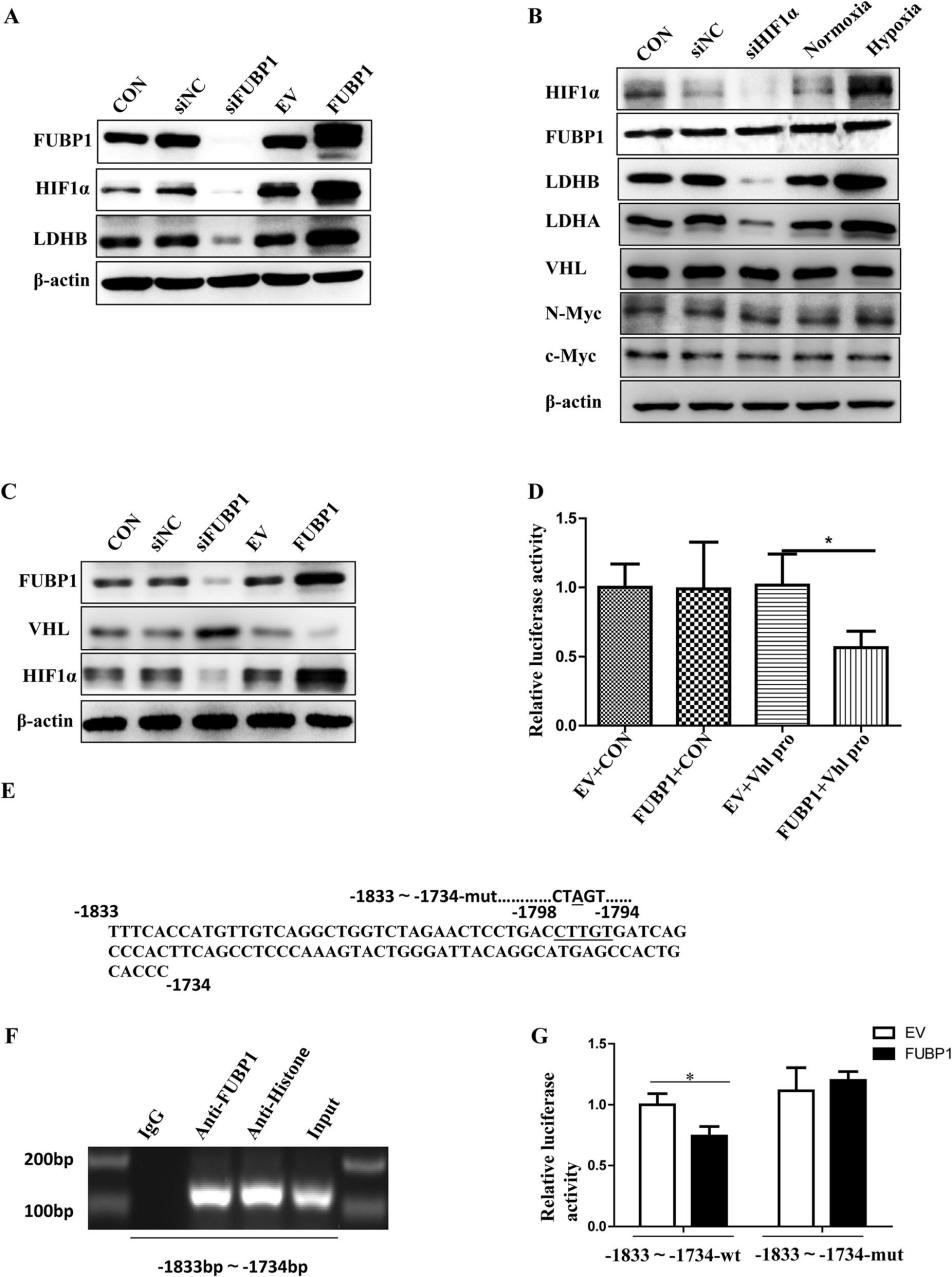
FUBP1 promotes LDHB expression by upregulating HIF1α. (**a**) SH-EP cells were infected with viruses expressing FUBP1 or with siRNA to knock down FUBP1 for 72 h, followed by western blot analysis. β-Actin served as a loading control. (**b**) SH-EP cells were infected with siRNA to knock down HIF1α for 72 h or placed in a hypoxic incubator for one week, followed by western blot analysis. (**c**) SH-EP cells were infected with siRNA to knock down FUBP1 for 72 h or followed by western blot analysis of VHL and HIF1α protein levels. (**d**) SH-EP cells were infected with FUBP1-overexpressing plasmid or empty vector plasmid, Vhl promoter luciferase reporter plasmid and Renilla luciferase plasmid for 48 h, followed by fluorescence detection. Renilla luciferase served as the transfection control. (**e**) The candidate FUBP1 binding site, CTTGT (underlined), is shown in the − 1798 to − 1794 region of the Vhl promoter sequence. (**f**) Chip assays were performed to verify FUBP1 binding to the Vhl promoter. Lane 1: PCR product from immunoprecipitated by normal IgG; Lane 2: PCR product derived from immunoprecipitation by anti-FUBP1 antibody Lane 3: PCR product from immunoprecipitation by anti-Histone antibody; Lane 4: PCR product from input DNA. (**g**) SH-EP cells were infected with FUBP1-overexpressing plasmid or empty vector plasmid, wild-type Vhl promoter reporter (− 1833 bp~ − 1734 bp) and mutant reporter (the single T to A substitution) for 48 h, followed by fluorescence detection. Renilla luciferase served as the transfection control. Error bars represent the standard deviation (SD); one asterisk, *p* < 0.05; NS means no significant difference

We stained c-Myc on the NB TMA. The staining results were scored according to the H-Score system and then counted. The results showed that compared with GN, the expression of c-Myc was increased in GNB and NB (Additional file 4: Fig. S4A-B). However, there was no significant difference between GNB and NB (Additional file 4: Fig. S4B). Furthermore, we found a correlation between c-Myc and FUBP1 expression in NB TMA (R = 0.173, *p* = 0.03; Additional file 4: Fig. S4C). Previously, research showed that c-Myc was an important regulator of aerobic glycolysis [29]. First, we verified the effect of siRNAs on c-Myc knockdown in SH-EP cells (Additional file 4: Fig. S4E). Additional file 4: Fig. S4D shows that LDHB levels were relatively high even though c-Myc was knocked down in NB cells overexpressing FUBP1, suggesting that FUBP1 may regulate NB glycolysis partially through the c-Myc pathway. Recent evidence concerning the detailed molecular mechanism of aerobic glycolysis has focused on the two regulators of the Warburg effect (c-Myc and HIF1α) [29]. Therefore, we determined whether HIF1α plays a significant role in NB glycolysis. The interfering effect of siHIF1α is shown in Additional file 3: Fig. S3E. Fortunately, Additional file 4: Fig. S4D shows that LDHB expression was decreased in NB cells with HIF1α knockdown. Furthermore, we found that HIF1α and LDHB levels were increased in NB tissues compared to GNB tissues (Fig. 5f). Figure 5g shows a strong positive correlation between FUBP1 and HIF1α expression in NB frozen tissues. Likewise, HIF1α and LDHB levels were increased in NB cell lines compared to normal cell lines (Fig. 5h). Moreover, HIF1α and LDHB mRNA and protein levels were increased after FUBP1 overexpression and downregulated after FUBP1 interference in NB cells (Fig. 6a, Fig. 5a). We further found that LDHB was downregulated after interfering with HIF1α and upregulated after culture in a hypoxic incubator with overexpression of HIF1α (Fig. 6b). The interfering effects of siHIF1α are shown in Additional file 4: Fig. S4E. However, FUBP1, c-Myc N-Myc and VHL levels were not changed, and LDHA was increased. (Fig.6b) Furthermore, Additional file 4: Fig. S4D demonstrated that c-Myc played a crucial role in regulating glycolytic enzyme LDHB expression with affecting HIF1α in NB cells. FUBP1 could influence downstream LDHB level by partially regulating the crucial factor c-Myc. The interference of both c-Myc and HIF1α were proved to eliminate LDHB levels in NB cells, which indicated that FUBP1 might upregulate LDHB expression to enhance NB glycolysis through c-Myc and HIF1α pathway.

Previous studies showed that N-Myc (MYCN) could regulate glycolytic key enzymes including HK2, PKM, LDHA, resulting in enhanced glycolysis in NB [37]. Our data showed that compared to GN, N-Myc levels were significantly different in GNB or NB (Additional file 5: Fig. S5A). Therefore, we hypothesized whether FUBP1 might regulate N-Myc, or N-Myc regulated FUBP1 to enhance NB glycolysis. To verify the relationship between FUBP1 and N-Myc, we detected the expression of FUBP1 in the SH-EP cell overexpressed or silenced N-Myc; and the expression of N-Myc in the SH-EP cell overexpressed or silenced FUBP1. However, the results demonstrated that N-Myc could not regulate FUBP1 or LDHB levels; FUBP1 could not regulate N-Myc in the SH-EP cell (Additional file 5: Fig. S5B-C), which indicated that the pathway FUBP1 or N-Myc regulating NB glycolysis were mutually independent.

Collectively, FUBP1 regulated downstream LDHB expression via targeting c-Myc and HIF1α resulting in the enhanced NB glycolysis, which was independent of N-Myc.

### FUBP1 upregulated HIF1α by promoting HIF1α mRNA levels and inhibiting HIF1α degradation via binding to the VHL promoter

**Fig. 7:**
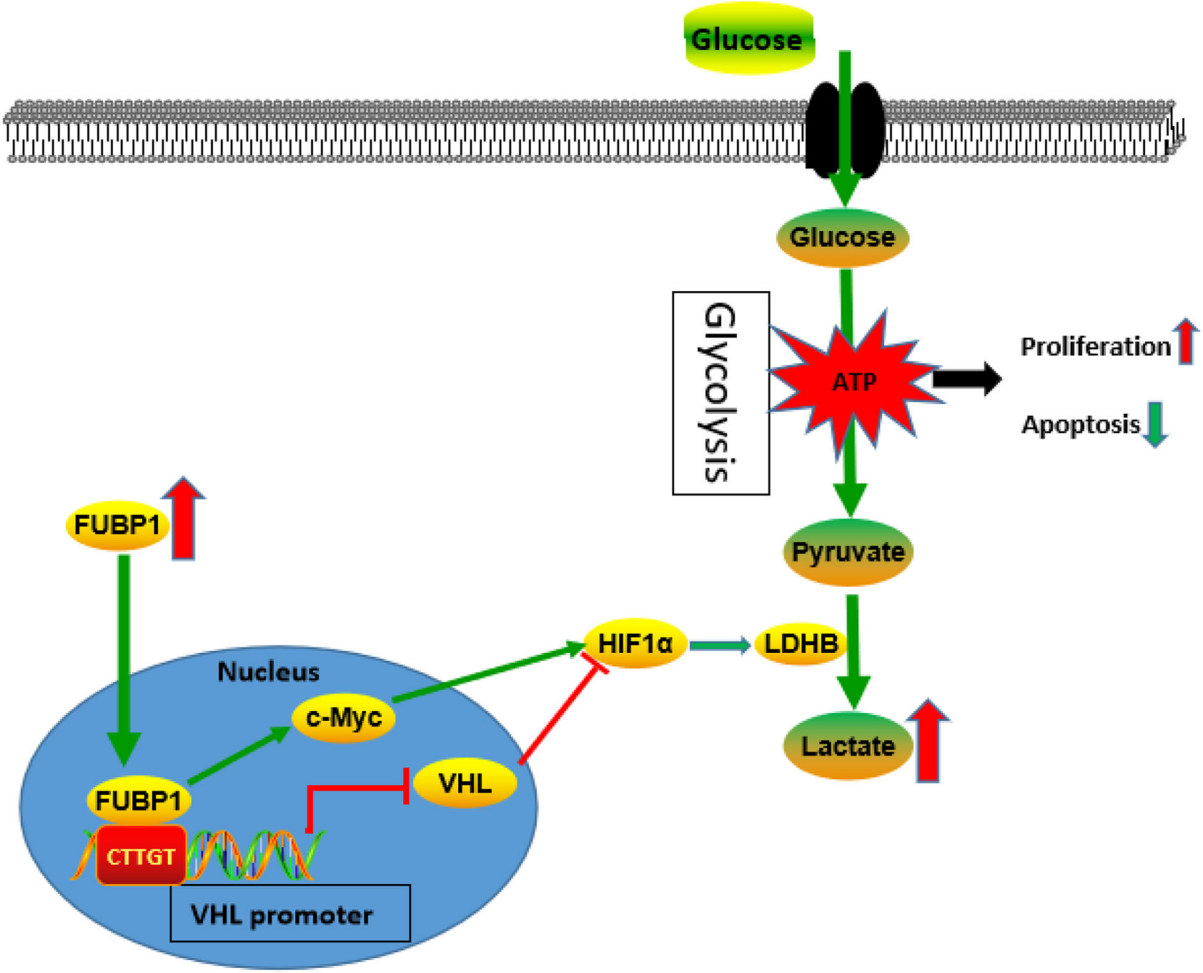
Simplified model depicting the pathway through how FUBP1 regulates NB growth

Previously, research regarding the elaborate regulatory mechanism of HIF1α protein concentrated on post-translational modification. VHL-mediated HIF1α degradation was clearly observed [38]. VHL, as the common HIF1α-E3 ligase, combined with HIF1α subunits resulted in their ubiquitination and degradation under normoxic conditions. Under normoxia, HIF1α was maintained at a low level via the hydroxylation by prolyl hydroxylase domain enzymes and the recognition and ubiquitination by the E3 ligase VHL, followed by proteasomal degradation. Hypoxia reduces the activity of prolyl hydroxylase domain enzymes, which led to the stabilization of HIFs and the initiation of HIF-dependent transcriptional expression [39]. Therefore, our data showed that VHL mRNA and protein levels were increased after interference with FUBP1 and downregulated after overexpression of FUBP1 in NB cells, suggesting that FUBP1 might regulate HIF1α protein levels through VHL (Fig. 6c and Additional file 7: Fig. S7A). To investigate whether VHL was transcriptionally regulated by FUBP1, promoter assays were undertaken. As shown in Fig.6d, luciferase reporters containing the full-length of the human VHL promoter were transiently transfected into SH-EP cells together with empty vector or overexpressing FUBP1 vector. Overexpression of FUBP1 inhibited activity of VHL promoter reporters. After carefully searching, we found one potential binding site (CTTGT), which is identical to the Usp29 FUSE binding site [17]. The candidate FUBP1 binding site, CTTGT (underlined), was shown in the − 1798 to − 1794 region of the Vhl promoter sequence (Fig. 6e). To confirm that FUBP1 can directly bind to the VHL promoter sequence (− 1833 bp ~ − 1734 bp), we performed ChiP assay using anti-FUBP1 antibody. As shown in Fig.6f, FUBP1 inhibited Vhl transcription through a FUBP1-binding site on the VHL promoter sequence (− 1833 bp ~ − 1734 bp). A single T- to A- substitution in the potential binding site abolished FUBP1 mediated down-regulation of VHL promoter reporter activity (Fig. 6g). Taken together, these results suggested that FUBP1 regulated downstream LDHB expression by upregulating HIF1α levels by binding to the VHL promoter and downregulating VHL levels (Fig. 7).

Notably, we found that FUBP1 might also bind to the HIF1α promoter to upregulate HIF1α mRNA levels (Additional file 6: Fig. S6A-B). Further research is needed to verify the precise FUBP1-binding site in the HIF1α promoter.

## Discussion

NB is one of the most common extracranial malignant solid tumours in children. This occult disease shows rapid progress, a high degree of malignancy and easy recurrence and is difficult to treat. However, its pathogenesis is not completely understood. The current study first demonstrated that the proportion of low N-Myc expression was much higher than the proportion of high N-Myc expression in NB pathological samples (Fig. 1a, c-d and Additional file 1: Fig. S1B). We speculated that other genetic alterations may play an important role in the development of NB. Then, the NB transcriptome microarray from the GEO database was re-analysed and showed that the tumour-associated transcription factor FUBP1 was significantly increased with NB malignancy (Fig. 1b-f and Additional file 1: Fig. S1C-D). Furthermore, we verified that there was a significant negative correlation between FUBP1 and the survival rate of patients (Fig. 1g). Moreover, FUBP1 promoted NB proliferation and inhibited apoptosis via promoting NB glycolysis. Mechanistically, these results demonstrated for the first time that abnormally elevated FUBP1 promotes NB glycolysis and growth by targeting both c-Myc and HIF1α. Here, we first identified that FUBP1 upregulated HIF1α mRNA expression by directly binding to the HIF1α promoter sequence and inhibited its degradation via downregulating VHL through targeting a FUBP1-binding site on the VHL promoter (Fig. 6a-g). These findings are the first to establish that abnormally increased FUBP1 plays an important role in NB, enrich our knowledge of the properties of FUBP1 and provide new indicators for the diagnosis and treatment of NB.

Some genetic alterations were clinically observed, such as MYCN amplification, ALK kinase activating mutations and some chromosome arm deletions, including 1p, 11q and 14q [40], in NB patients. Meanwhile, previous studies demonstrated that *CDH5*, *PAFAH1B1* and *NME1* were strongly associated with NB patient outcome [41]. The oncoprotein N-Myc promoted cell proliferation, differentiation and malignant transformation in NB [23]. Although genomic amplification of MYCN was the common genetic aberration consistently associated with poor prognosis and was deemed the vital marker for tumour recurrence and malignancy in NB, it was only detected in less than 30% of all NB cases [7]. In the NB TMA, the percentage of N-Myc low expression in NB tissues (*n* = 65) was 63.1% (Fig. 1a and c-d), suggesting that other genetic alterations should be explored to explain the phenomenon in the development of NB. Furthermore, the NB transcriptome data from the GEO database were analysed, and we found that FUBP1 mRNA was augmented with the rise in the International Neuroblastoma Staging System (INSS) stage (Additional file 1: Fig. S1D). FUBP1 is highly expressed in a variety of tumour tissues and cell lines [10, 42, 43]. However, the expression of FUBP1 in neurogenic tumours is still unclear, and its expression level and effect in neuroblastoma should be further studied. We found that there was almost no expression of FUBP1 in GN, in which malignancy was lowest and differentiation was highest. FUBP1 was commonly expressed in NB, which was the most malignant and undifferentiated NB (Fig. 1f & Additional file 1: Fig. S1D). In the present study, we collected the follow-up data of 70 patients and found that the survival rate of patients with high FUBP1 levels was significantly lower than that of patients with low FUBP1 levels (Fig. 1h). These results suggest that FUBP1 plays an important role in NB and is expected to be a prognostic marker for NB. To confirm our theory, we are continuing to collect the remaining patient follow-up information and undertaking an ongoing follow-up of the currently surviving patients.

In normal cells, c-Myc is induced upon growth factor stimulation, whereas it is constitutively highly expressed in transformed cells. Some degree of c-Myc overexpression is estimated to occur in 70% of human tumours [44]. The oncoprotein c-Myc is known to be involved in the regulation of aerobic glycolysis in tumour cells. Previously, evidence has demonstrated that c-Myc upregulates metabolic enzymes such as HK2, PKM2 and LDHA [45]. A recent study showed that LDHB was significantly upregulated in diffuse large B-cell lymphoma associated with high c-Myc expression [46], which reveals that LDHB can be regulated by c-Myc. Interestingly, we found that FUBP1 could upregulate LDHB, enhancing glycolysis partially via targeting c-Myc. Likewise, N-Myc can activate target genes to influence aerobic glycolysis [47]. However, we found that LDHB was not regulated by N-Myc in NB. Recent studies have demonstrated that c-Myc and HIF1α are the two major regulators of the Warburg effect. Moreover, the regulatory pathway of aerobic glycolysis is interactional [29–32]. Interestingly, our data demonstrated that c-Myc could influence expression of the glycolytic enzyme LDHB with affecting HIF1α in NB (Additional file 4: Fig. S4D).

As an important transcription factor, HIF1α regulates the expression of the key glycolytic enzymes involved in the reaction to hypoxia, such as HK2, aldolase A, pyruvate kinase M, LDHA and phosphoglycerate kinase 1 [24–28], while the detailed mechanism is still unclear. Our data showed that FUBP1 could upregulate LDHB to enhance glycolysis via targeting HIF1α, independent of several key glycolytic enzymes previously described (Fig. 5a-f). Previous studies demonstrated that LDHA was the prominent isoform in neuroblastoma [36]. In our study, the data indicated that LDHA was poorly associated with survival rate (Additional file 3: Fig. S3C). While we analyzed the relationship between LDHA levels in the neuroblastoma which pathological histotype is NB not GNB or GN and survival outcome of those NB patients, the results showed that LDHA was associated with survival rate (Additional file 3: Fig. S3D). Meanwhile, previous study showed that LDHA inhibition decreases neuroblastoma growth independent of aerobic glycolysis, which indicated LDHA was not the important enzyme in the metabolism of aerobic glycolysis in NB [36]. Therefore, we focus on LDHB to explain why FUBP1 could promote NB glycolysis.

Previous studies have shown that in addition to being regulated by ambient oxygen levels, HIF1α protein levels are also affected by a variety of other factors, such as Src, K-ras, MSF-A, and BRCA1 [48–50]. These factors can promote HIF1α expression by reducing its degradation and activating its transcriptional activity [50]. Furthermore, PTEN can downregulate HIF1α protein through the PI3K-AKT pathway [51]. In addition, mutations in the SDH and FH genes lead to an increase in succinic acid and a decrease in α-ketoglutarate, which in turn inhibits proline hydroxylase to inhibit ubiquitination of HIF1α [52]. As the common HIF1α-E3 ligase, VHL combined with HIF1α subunits resulted in their ubiquitination and degradation under normoxic conditions [38]. Notably, the mechanism of VHL protein-protein regulation or post-translational modification has been clearly clarified, while the VHL transcription factors are still unclear. In this study, we found that FUBP1 upregulated HIF1α transcriptional expression (Additional file 6: Fig. S6A-B) and downregulated VHL to inhibit HIF1α degradation (Fig. 6c). Here, FUBP1 directly bound to the promoter sequence of the VHL gene to inhibit its expression (Fig. 6d-g, Additional file 7: Fig. S7B). Thus, it could provide a new theoretical basis for further study of the biological function of FUBP1 and the regulatory mechanism of HIF1α. Likewise, this molecule could be a novel diagnostic and prognostic indicator for NB and other tumours regulated by HIF1α.

The reasons for the abnormal increase of FUBP1 in NB should be deserving of attention. Previous studies have found that FUBP1 has mutations in oligodendroglioma, and its mutation is similar with the TP53 gene. The exon mutation is predominant [53], and 5 cases of FUBP1 mutations were detected in 30 patients [54]. We suspected that there might be a mutation of FUBP1 in NB that causes it to increase abnormally. We selected 5 NB paraffin-embedded tissue samples with high levels of FUBP1, 6 NB frozen pNT tissue samples and the 5 most malignant NB cells to sequence exons 5–14 of the Fubp1 gene. The results showed that no possible mutation sites of FUBP1 were found in NB tissues and cell lines (data not shown). A previous study showed that miR-16 played a role in anti-tumour apoptosis in breast cancer and gastric cancer by downregulating FUBP1 [12]. Furthermore, the PI3K/AKT/mTOR pathway contributed to FUBP1 stabilization through inhibiting caspase3/7 activity in hepatocellular carcinoma cells [55]. Previous studies have shown that the PI3K/AKT/mTOR signalling cascade is overactivated in neuroblastoma [56]. Whether the abnormal increase in FUBP1 is regulated by the miR-16 or PI3K/AKT/mTOR pathway, further research is needed in the future.

In summary, we identified a critical role of FUBP1, another important oncogene in the development of NB independent of N-Myc. Abnormally elevated FUBP1 enhancing glycolysis might be the reason for the fast growth of NB. Mechanistically, the FUBP1-HIF1α-LDHB axis might potentially be a novel target for the diagnosis and treatment of NB.

## Conclusions

Taken together, our data demonstrated that FUBP1, another important oncogene independent of N-Myc, was upregulated and significantly correlated with poor clinical survival in NB. FUBP1 could promote cell proliferation and inhibit apoptosis via enhancing glycolysis and ATP production in NB cells. Moreover, FUBP1 was a transcription factor to regulate HIF1α expression by directly binding to VHL promoter sequence, which increased glycolytic enzyme LDHB expression to promote glycolysis and growth of NB. FUBP1 could be a novel therapeutic marker and target for diagnosis in NB.

## Supplementary information

Additional file 1: **Figure S1.** FUBP1 was increased in NB tissue. (A) The arrangement of the NB tissue microarray. (B) Immunohistochemistry staining of n-Myc. (C) Immunohistochemistry staining of FUBP1. (D) The analysis of NB transcriptome data from GEO database. (E) Western blot analysis of the effects of overexpressing or interfering FUBP1.(F) qPCR analysis of plasmid overexpressing FUBP1 or with siRNA to knock down FUBP1. (G) Western blot analysis of the interfering effects of siRNAs. (TIF 5709 kb)

Additional file 2: **Figure S2.** FUBP1 inhibited NB cell apoptosis. (A) Flow cytometry analysis of SK-N-BE(2) cells apoptosis. (B) Statistical analysis of apoptotic cells rate in SK-N-BE(2) cell. (C) Flow cytometry analysis of SH-EP cells apoptosis. (D) Statistical analysis of apoptotic cells rate in SH-EP cell. (E) Flow cytometry analysis of apoptotic rate of SK-N-BE(2) cells incubated with hypoxia. (F) Statistical analysis of apoptotic cells rate in SK-N-BE(2) cell. (TIF 2777 kb)

Additional file 3: **Figure S3.** LDHA was correlated with NB survival. (A) Immunohistochemistry staining of LDHA. (B) Statistical analysis of of LDHA. (C) Survival of NB patients with high expressed or with low expressed LDHA. (D) Survival of NB patients with high expressed or with low expressed LDHA. (E) Correlation analysis of LDHA and FUBP1 expression. (TIF 3230 kb)

Additional file 4: **Figure S4.** FUBP1 influenced c-Myc to upregulate LDHB. (A) Immunohistochemistry staining of c-Myc. (B) Statistical analysis of c-Myc. (C) Correlation analysis of c-Myc and FUBP1 expression. (D) Western blot analysis of interfering effects of c-Myc. (E) Western blot analysis of LDHB. (TIF 3986 kb)

Additional file 5: **Figure S5.** FUBP1 regulated LDHB independent of N-Myc. (A) Statistical analysis of n-Myc in TMA. (B) Western blot analysis of FUBP1, c-Myc and LDHB levels. (C) Western blot analysis of interfering effects of N-Myc. (D) Western blot analysis of N-Myc. (TIF 2302 kb)

Additional file 6: **Figure S6.** FUBP1 could regulate HIF1α mRNA. (A) qPCR analysis of Hif1α mRNA. (B) Luciferase reporter analysis of Hif1α promoter. (TIF 1399 kb)

Additional file 7: **Figure S7.** FUBP1 bound to VHL promoter. (A) qPCR analysis of VHL mRNA. (B) Chip assays of VHL promoter other sequences (-1434bp∼-1326bp and -545bp∼-433bp). (TIF 1631 kb)

Additional file 8: **Table S1.** Clinical and biological characteristics in NB tumor samples. (PDF 158 kb)

## Data Availability

All data generated or analysed during this study are included in this published article.

## Abbreviations

FUBP1: far upstream element binding protein 1
HIF1α: Hypoxia-inducile factor 1-alpha
IHC: Immunohistochemistry
LDHB: Lactate dehydrogenase isoform B
NB: Neuroblastoma
pNTs: peripheral neuroblastic tumors
TMA: Tissue microassay
VHL: Von Hippel-Lindau

## References

[1] Pinto NR (2015). Advances in risk classification and treatment strategies for neuroblastoma. J Clin Oncol.

[2] Shimada H (1999). The international neuroblastoma pathology classification (the Shimada system). Cancer.

[3] Yang T (2017). Surgical management and outcomes of ganglioneuroma and ganglioneuroblastoma-intermixed. Pediatr Surg Int.

[4] Mora J, Cheung NK, Gerald WL (2001). Genetic heterogeneity and clonal evolution in neuroblastoma. Br J Cancer.

[5] Huang M, Weiss WA (2013). Neuroblastoma and MYCN. Cold Spring Harb Perspect Med.

[6] Wakamatsu Y, Watanabe Y, Nakamura H, Kondoh H (1997). Regulation of the neural crest cell fate by N-myc: promotion of ventral migration and neuronal differentiation. Development.

[7] Brodeur GM (2003). Neuroblastoma: biological insights into a clinical enigma. Nat Rev Cancer.

[8] Schwab M, Westermann F, Hero B, Berthold F (2003). Neuroblastoma: biology and molecular and chromosomal pathology. Lancet Oncol.

[9] Masserot C (2016). WT1 expression is inversely correlated with MYCN amplification or expression and associated with poor survival in non-MYCN-amplified neuroblastoma. Mol Oncol.

[10] Malz M (2009). Overexpression of far upstream element binding proteins: a mechanism regulating proliferation and migration in liver cancer cells. Hepatology.

[11] Wang Xiao-tong, Xia Qiu-yuan, Ye Sheng-bing, Wang Xuan, Li Rui, Fang Ru, Shi Shan-shan, Zhang Ru-song, Tan Xiao, Chen Jie-yu, Sun Ke, Teng Xiao-dong, Ma Heng-hui, Lu Zhen-feng, Zhou Xiao-jun, Rao Qiu (2018). RNA sequencing of Xp11 translocation-associated cancers reveals novel gene fusions and distinctive clinicopathologic correlations. Modern Pathology.

[12] Venturutti L (2016). MiR-16 mediates trastuzumab and lapatinib response in ErbB-2-positive breast and gastric cancer via its novel targets CCNJ and FUBP1. Oncogene.

[13] Khageh Hosseini S (2017). Camptothecin and its analog SN-38, the active metabolite of irinotecan, inhibit binding of the transcriptional regulator and oncoprotein FUBP1 to its DNA target sequence FUSE. Biochem Pharmacol.

[14] Singer S (2009). Coordinated expression of stathmin family members by far upstream sequence element-binding protein-1 increases motility in non-small cell lung cancer. Cancer Res.

[15] Braddock DT, Louis JM, Baber JL, Levens D, Clore GM (2002). Structure and dynamics of KH domains from FBP bound to single-stranded DNA. Nature.

[16] Duncan R (1994). A sequence-specific, single-strand binding protein activates the far upstream element of c-myc and defines a new DNA-binding motif. Genes Dev.

[17] Liu J (2011). JTV1 co-activates FBP to induce USP29 transcription and stabilize p53 in response to oxidative stress. EMBO J.

[18] Wang, J. *et al.* A novel long intergenic noncoding RNA indispensable for the cleavage of mouse two-cell embryos. *EMBO Rep* **17**, 1452–1470, doi:10.15252/embr.201642051 (2016). 10.15252/embr.201642051 PMC5048373 27496889

[19] Sun Y (2017). The long noncoding RNA SNHG1 promotes tumor growth through regulating transcription of both local and distal genes. Oncogene.

[20] Zhong Q (2018). The RARS-MAD1L1 fusion gene induces Cancer stem cell-like properties and therapeutic resistance in nasopharyngeal carcinoma. Clin Cancer Res.

[21] Walenta S (2000). High lactate levels predict likelihood of metastases, tumor recurrence, and restricted patient survival in human cervical cancers. Cancer Res.

[22] Gatenby RA, Gillies RJ (2004). Why do cancers have high aerobic glycolysis?. Nat Rev Cancer.

[23] Meyer N, Penn LZ (2008). Reflecting on 25 years with MYC. Nat Rev Cancer.

[24] Cheung EC, Ludwig RL, Vousden KH (2012). Mitochondrial localization of TIGAR under hypoxia stimulates HK2 and lowers ROS and cell death. Proc Natl Acad Sci U S A.

[25] Grandjean G (2016). Definition of a novel feed-forward mechanism for glycolysis-HIF1alpha signaling in hypoxic tumors highlights aldolase a as a therapeutic target. Cancer Res.

[26] Wang HJ (2014). JMJD5 regulates PKM2 nuclear translocation and reprograms HIF-1alpha-mediated glucose metabolism. Proc Natl Acad Sci U S A.

[27] Harris AL (2015). A new Hydroxy metabolite of 2-Oxoglutarate regulates metabolism in hypoxia. Cell Metab.

[28] Velpula KK, Bhasin A, Asuthkar S, Tsung AJ (2013). Combined targeting of PDK1 and EGFR triggers regression of glioblastoma by reversing the Warburg effect. Cancer Res.

[29] Gordan JD, Thompson CB, Simon MC (2007). HIF and c-Myc: sibling rivals for control of cancer cell metabolism and proliferation. Cancer Cell.

[30] Lin XW, Tang L, Yang J, Xu WH (2016). HIF-1 regulates insect lifespan extension by inhibiting c-Myc-TFAM signaling and mitochondrial biogenesis. Biochim Biophys Acta.

[31] Oh ET, Kim CW, Kim HG, Lee JS, Park HJ (2017). Brusatol-mediated inhibition of c-Myc increases HIF-1alpha degradation and causes cell death in colorectal Cancer under hypoxia. Theranostics.

[32] Chen C (2013). C-Myc enhances colon cancer cell-mediated angiogenesis through the regulation of HIF-1alpha. Biochem Biophys Res Commun.

[33] Qing G (2012). ATF4 regulates MYC-mediated neuroblastoma cell death upon glutamine deprivation. Cancer Cell.

[34] Lemkuil BP (2011). Isoflurane neurotoxicity is mediated by p75NTR-RhoA activation and actin depolymerization. Anesthesiology.

[35] Zhang T (2017). Deficiency of pigment epithelium-derived factor in nasopharyngeal carcinoma cells triggers the epithelial-mesenchymal transition and metastasis. Cell Death Dis.

[36] Dorneburg C (2018). LDHA in neuroblastoma is associated with poor outcome and its depletion decreases neuroblastoma growth independent of aerobic glycolysis. Clin Cancer Res.

[37] Chen QR (2010). Global genomic and proteomic analysis identifies biological pathways related to high-risk neuroblastoma. J Proteome Res.

[38] Lee JH, Elly C, Park Y, Liu YC (2015). E3 ubiquitin ligase VHL regulates hypoxia-inducible factor-1alpha to maintain regulatory T cell stability and suppressive capacity. Immunity.

[39] Semenza GL (2011). Oxygen sensing, homeostasis, and disease. N Engl J Med.

[40] Maris JM, Hogarty MD, Bagatell R, Cohn SL (2007). Neuroblastoma. Lancet.

[41] Skrzypski M (2008). Three-gene expression signature predicts survival in early-stage squamous cell carcinoma of the lung. Clin Cancer Res.

[42] Rabenhorst U (2009). Overexpression of the far upstream element binding protein 1 in hepatocellular carcinoma is required for tumor growth. Hepatology.

[43] Malz M (2014). Overexpression of far upstream element (FUSE) binding protein (FBP)-interacting repressor (FIR) supports growth of hepatocellular carcinoma. Hepatology.

[44] Nilsson JA, Cleveland JL (2003). Myc pathways provoking cell suicide and cancer. Oncogene.

[45] Miller DM, Thomas SD, Islam A (2012). Muench, D. & Sedoris, K. c-Myc and cancer metabolism. Clin Cancer Res.

[46] Xu-Monette ZY (2016). Clinical and biologic significance of MYC genetic mutations in De novo diffuse large B-cell lymphoma. Clin Cancer Res.

[47] Beltran H (2014). The N-myc oncogene: maximizing its targets, regulation, and therapeutic potential. Mol Cancer Res.

[48] Chao C (2007). Constitutively active CCK2 receptor splice variant increases Src-dependent HIF-1 alpha expression and tumor growth. Oncogene.

[49] Eisinger-Mathason TS (2013). Hypoxia-dependent modification of collagen networks promotes sarcoma metastasis. Cancer Discov.

[50] Amir S, Wang R, Matzkin H, Simons JW, Mabjeesh NJ (2006). MSF-A interacts with hypoxia-inducible factor-1alpha and augments hypoxia-inducible factor transcriptional activation to affect tumorigenicity and angiogenesis. Cancer Res.

[51] Requejo-Aguilar R (2014). PINK1 deficiency sustains cell proliferation by reprogramming glucose metabolism through HIF1. Nat Commun.

[52] Selak MA (2005). Succinate links TCA cycle dysfunction to oncogenesis by inhibiting HIF-alpha prolyl hydroxylase. Cancer Cell.

[53] Bettegowda C (2011). Mutations in CIC and FUBP1 contribute to human oligodendroglioma. Science.

[54] Cahill DP, Louis DN, Cairncross JG (2015). Molecular background of oligodendroglioma: 1p/19q, IDH, TERT, CIC and FUBP1. CNS Oncol.

[55] Samarin J (2016). PI3K/AKT/mTOR-dependent stabilization of oncogenic far-upstream element binding proteins in hepatocellular carcinoma cells. Hepatology.

[56] Westhoff MA (2014). A critical evaluation of PI3K inhibition in glioblastoma and neuroblastoma therapy. Mol Cell Ther.

