# Supplementary figures and images for: FUBP1 promotes neuroblastoma proliferation via enhancing glycolysis-a new possible marker of malignancy for neuroblastoma
Source: J Exp Clin Cancer Res. 2019 Sep 11;38:400. doi: 10.1186/s13046-019-1414-6 (PMC6737630; doi:10.1186/s13046-019-1414-6)

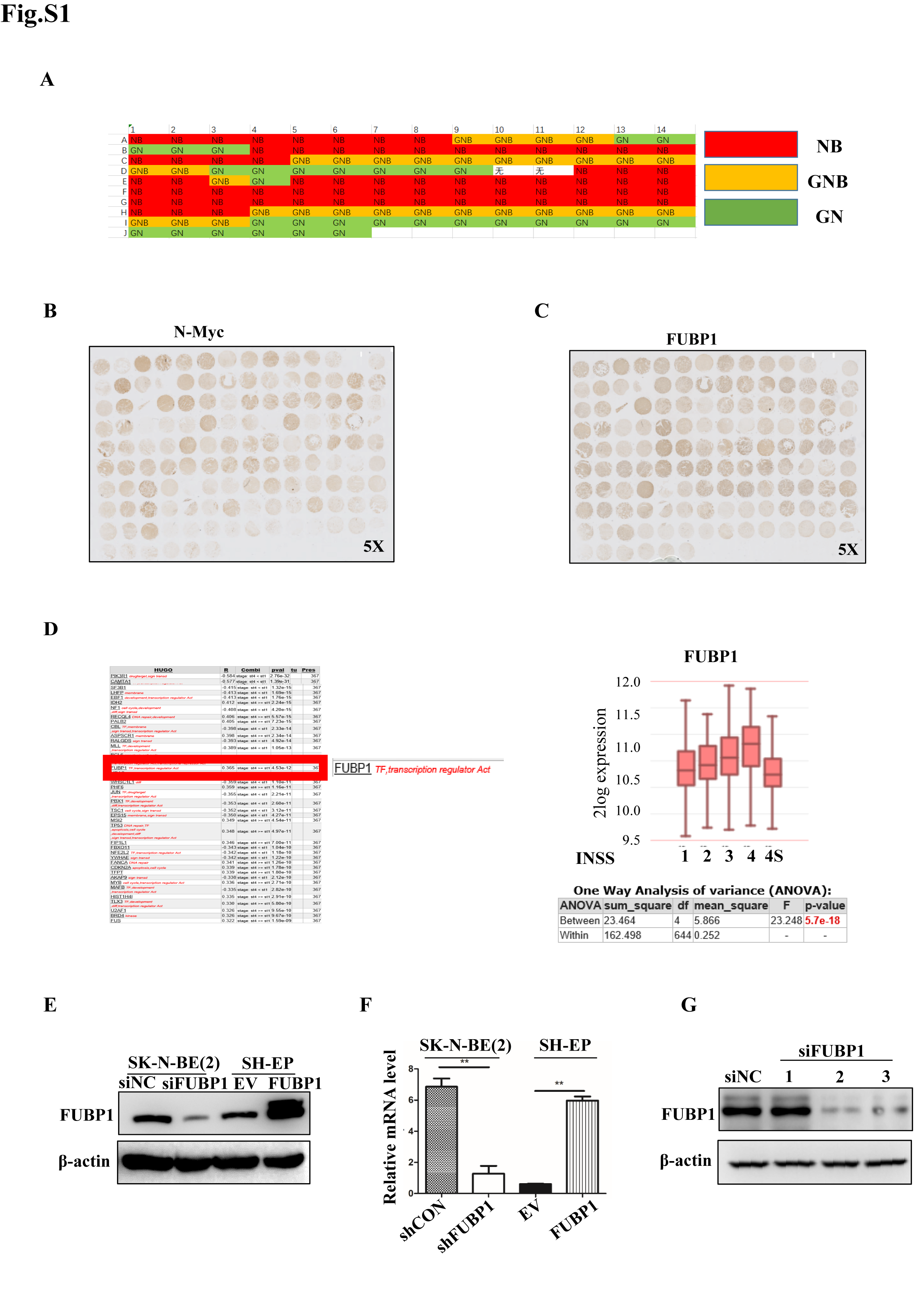

Supplement: Supplementary file 1 — Figure S1. FUBP1 was increased in NB tissue. (A) The arrangement of the NB tissue microarray. (B) Immunohistochemistry staining of n-Myc. (C) Immunohistochemistry staining of FUBP1. (D) The analysis of NB transcriptome data from GEO database. (E) Western blot analysis of the effects of overexpressing or interfering FUBP1.(F) qPCR analysis of plasmid overexpressing FUBP1 or with siRNA to knock down FUBP1. (G) Western blot analysis of the interfering effects of siRNAs. (TIF 5709 kb) [file 13046_2019_1414_MOESM1_ESM.tif]

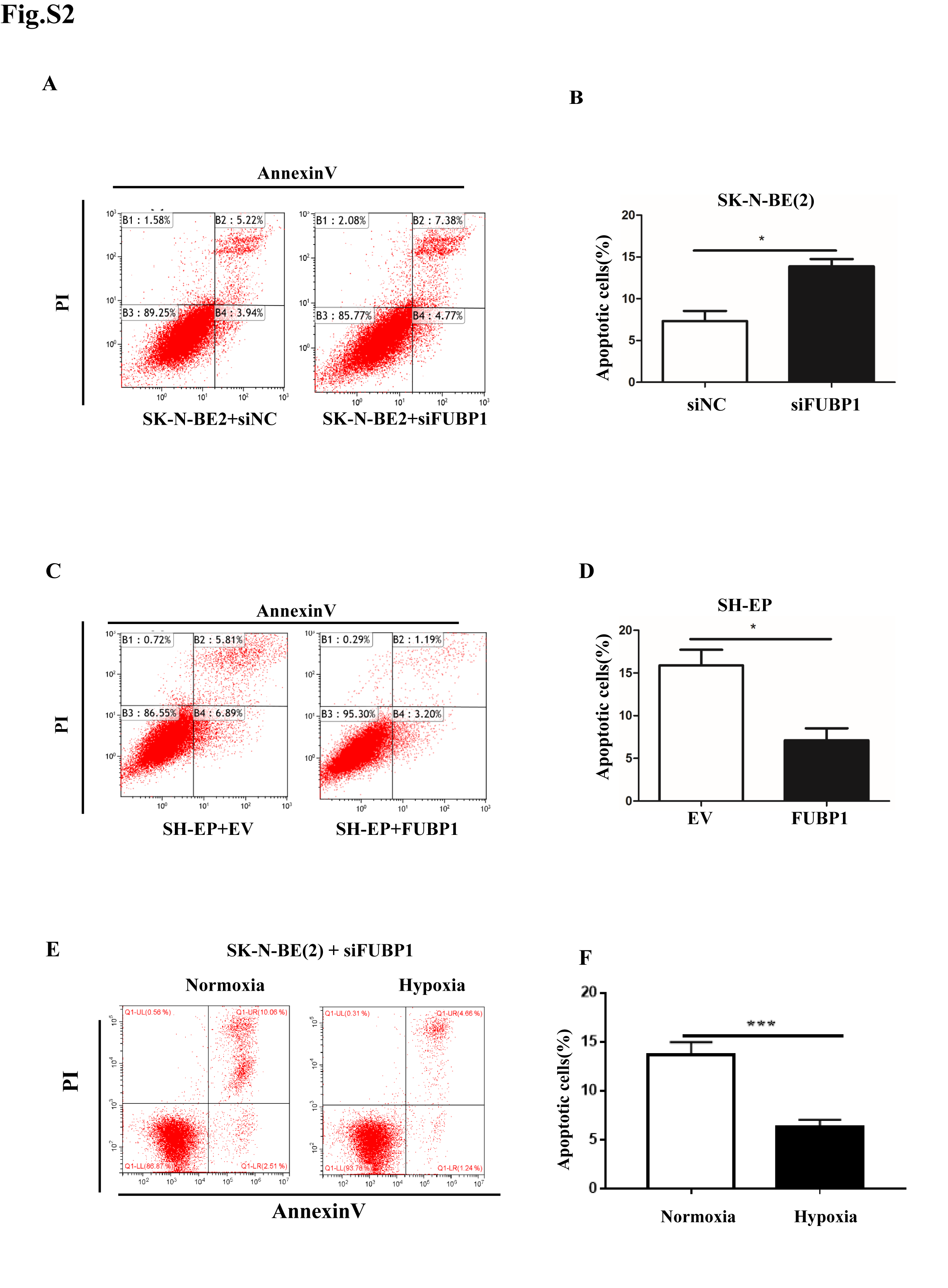

Supplement: Supplementary file 2 — Figure S2. FUBP1 inhibited NB cell apoptosis. (A) Flow cytometry analysis of SK-N-BE(2) cells apoptosis. (B) Statistical analysis of apoptotic cells rate in SK-N-BE(2) cell. (C) Flow cytometry analysis of SH-EP cells apoptosis. (D) Statistical analysis of apoptotic cells rate in SH-EP cell. (E) Flow cytometry analysis of apoptotic rate of SK-N-BE(2) cells incubated with hypoxia. (F) Statistical analysis of apoptotic cells rate in SK-N-BE(2) cell. (TIF 2777 kb) [file 13046_2019_1414_MOESM2_ESM.tif]

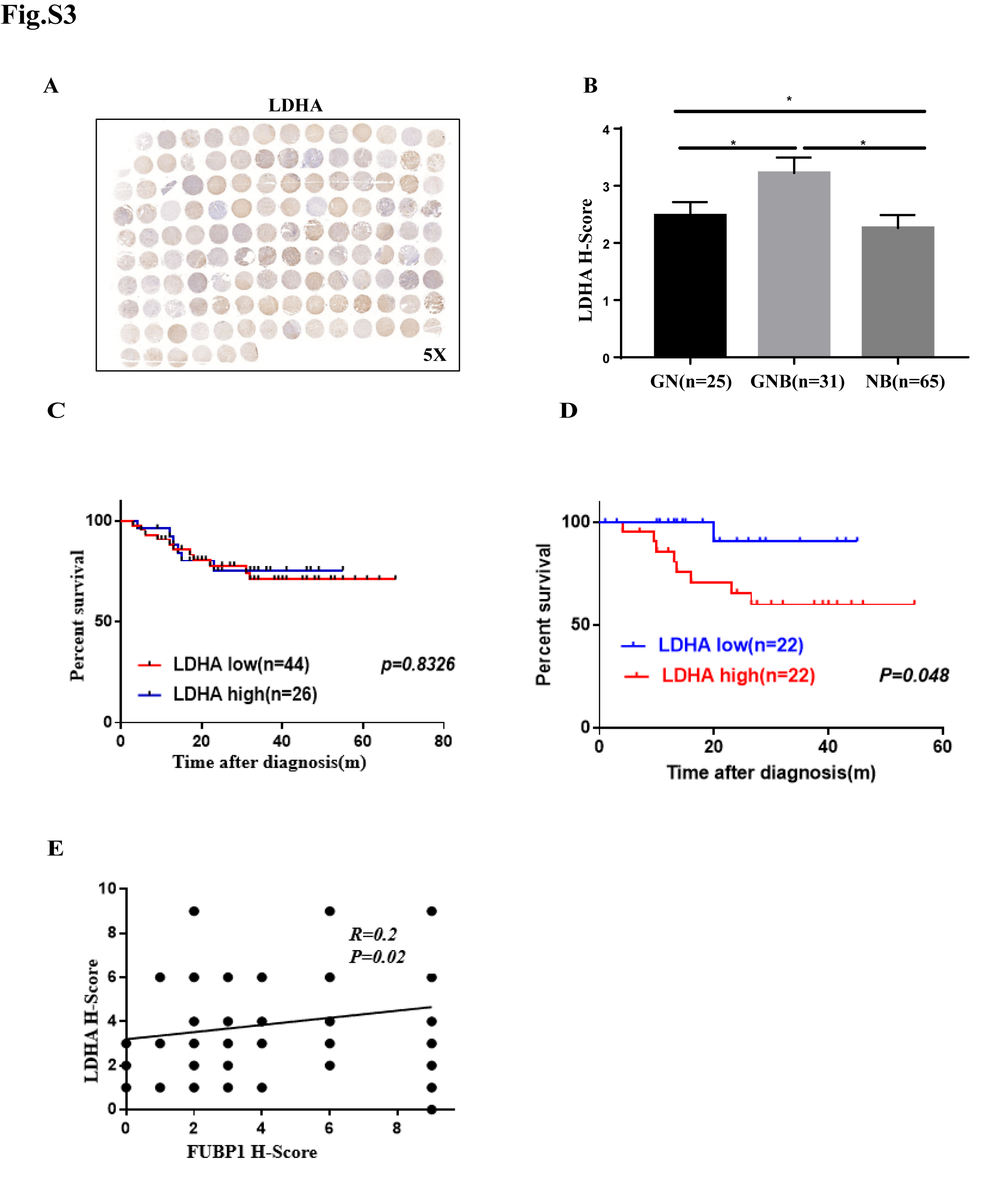

Supplement: Supplementary file 3 — Figure S3. LDHA was correlated with NB survival. (A) Immunohistochemistry staining of LDHA. (B) Statistical analysis of of LDHA. (C) Survival of NB patients with high expressed or with low expressed LDHA. (D) Survival of NB patients with high expressed or with low expressed LDHA. (E) Correlation analysis of LDHA and FUBP1 expression. (TIF 3230 kb) [file 13046_2019_1414_MOESM3_ESM.tif]

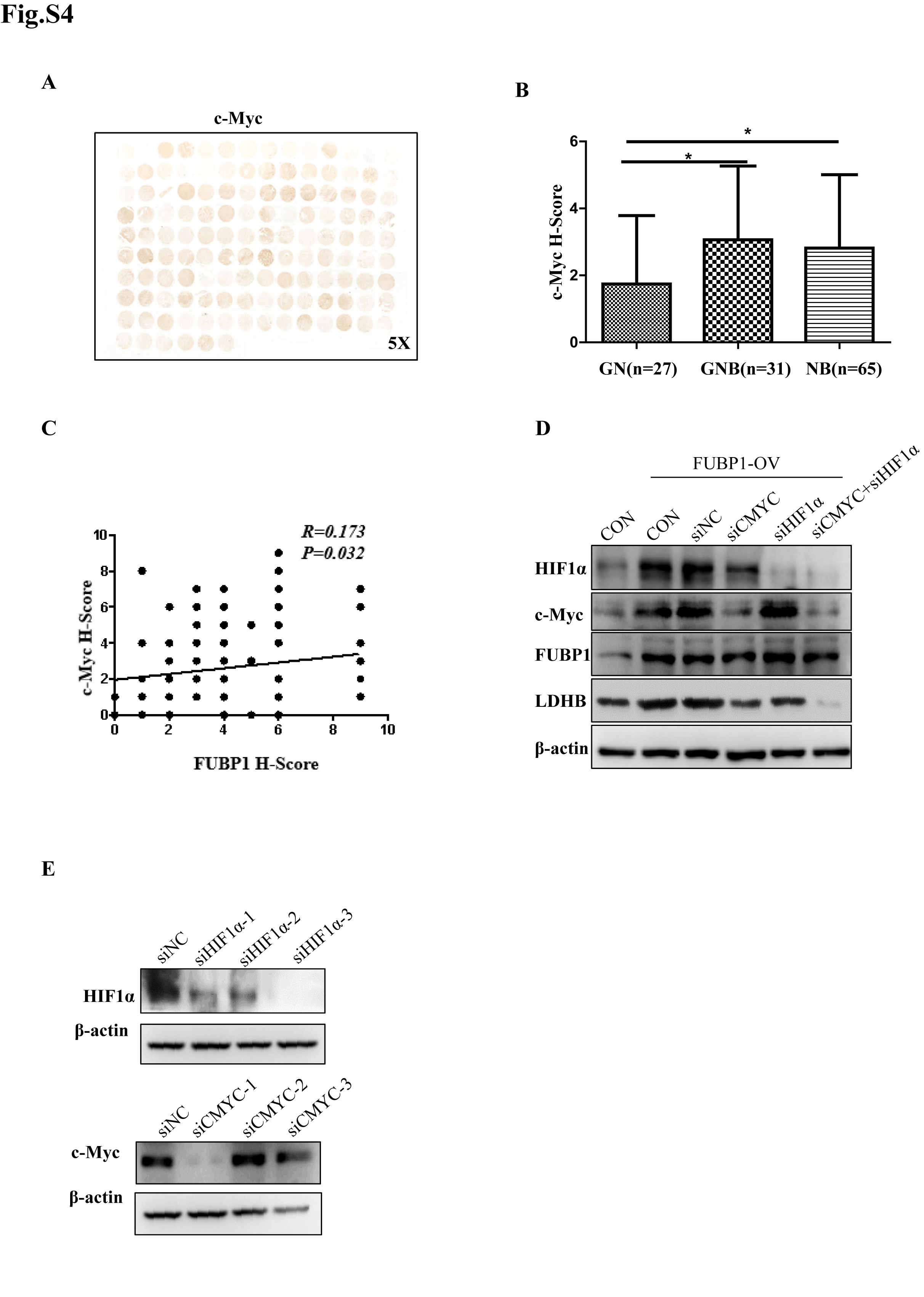

Supplement: Supplementary file 4 — Figure S4. FUBP1 influenced c-Myc to upregulate LDHB. (A) Immunohistochemistry staining of c-Myc. (B) Statistical analysis of c-Myc. (C) Correlation analysis of c-Myc and FUBP1 expression. (D) Western blot analysis of interfering effects of c-Myc. (E) Western blot analysis of LDHB. (TIF 3986 kb) [file 13046_2019_1414_MOESM4_ESM.tif]

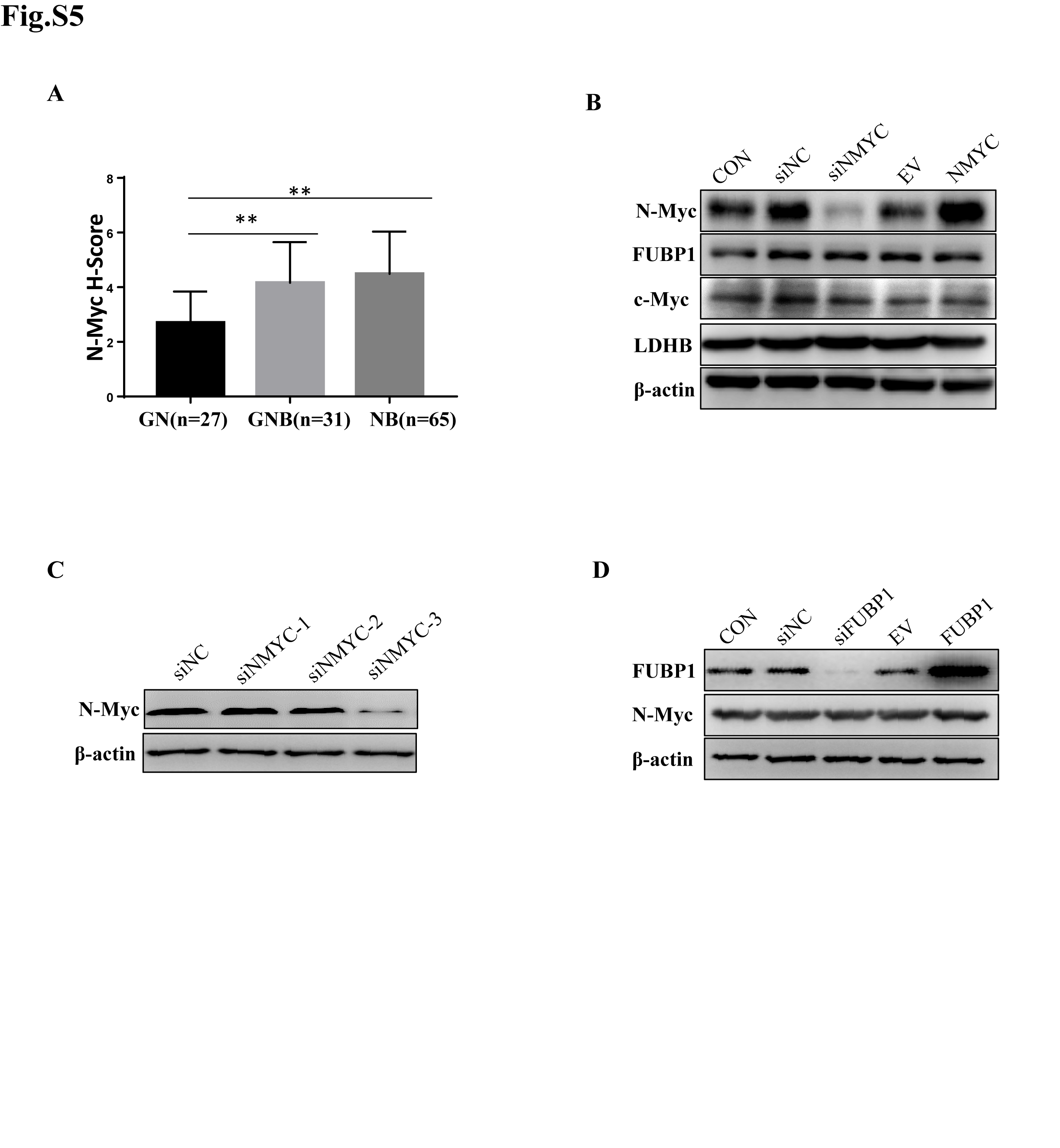

Supplement: Supplementary file 5 — Figure S5. FUBP1 regulated LDHB independent of N-Myc. (A) Statistical analysis of n-Myc in TMA. (B) Western blot analysis of FUBP1, c-Myc and LDHB levels. (C) Western blot analysis of interfering effects of N-Myc. (D) Western blot analysis of N-Myc. (TIF 2302 kb) [file 13046_2019_1414_MOESM5_ESM.tif]

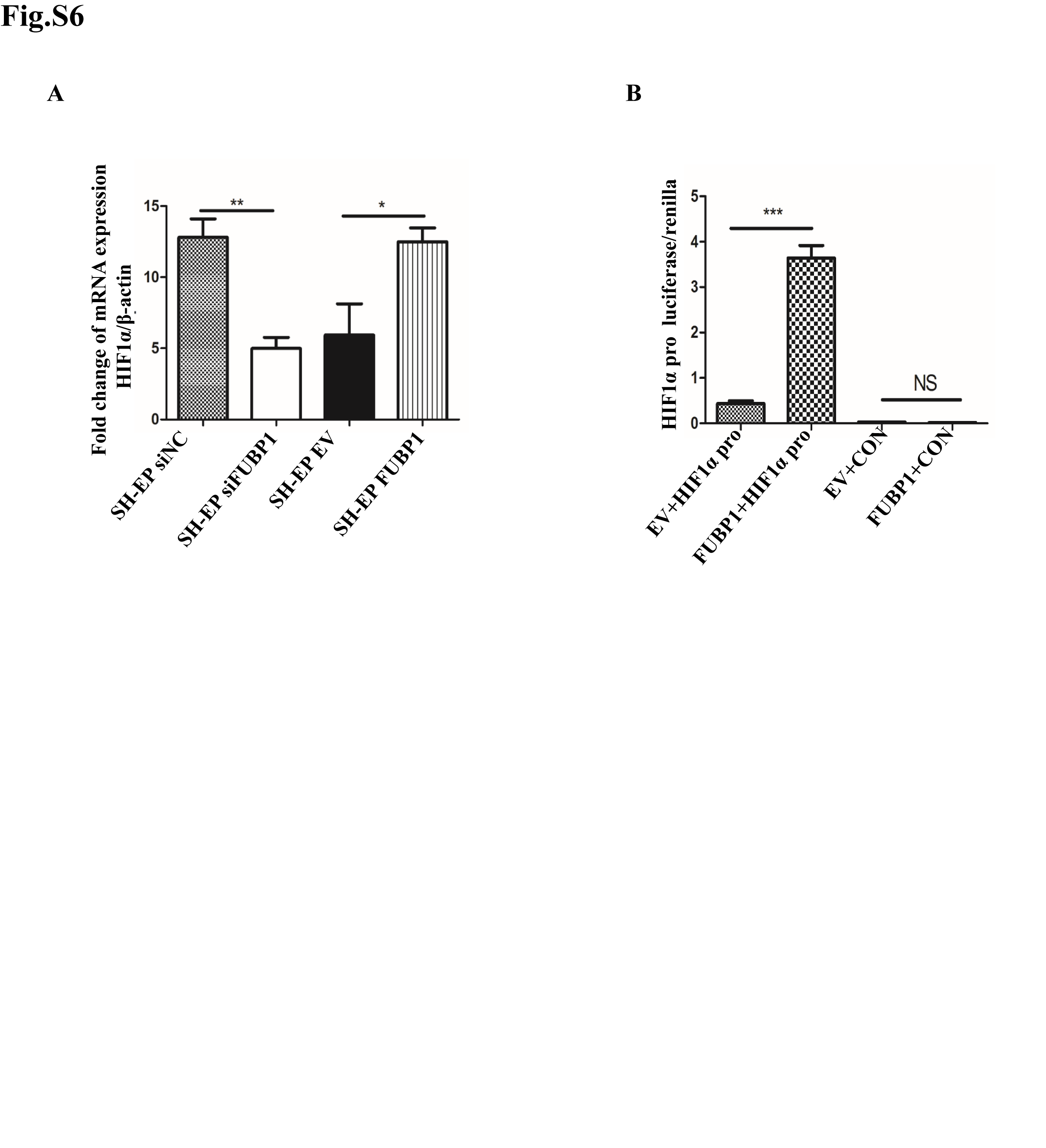

Supplement: Supplementary file 6 — Figure S6. FUBP1 could regulate HIF1α mRNA. (A) qPCR analysis of Hif1α mRNA. (B) Luciferase reporter analysis of Hif1α promoter. (TIF 1399 kb) [file 13046_2019_1414_MOESM6_ESM.tif]

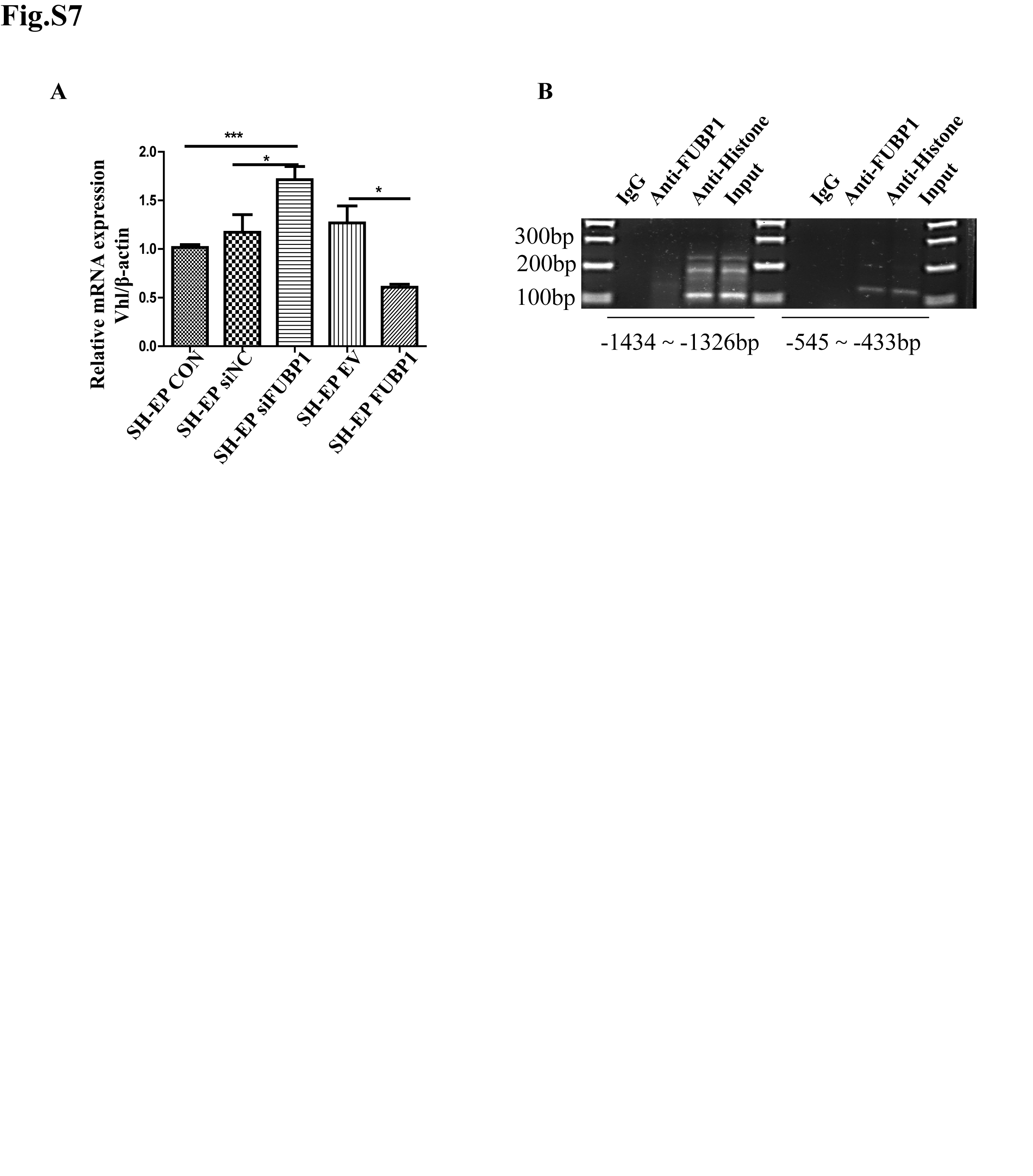

Supplement: Supplementary file 7 — Figure S7. FUBP1 bound to VHL promoter. (A) qPCR analysis of VHL mRNA. (B) Chip assays of VHL promoter other sequences (-1434bp∼-1326bp and -545bp∼-433bp). (TIF 1631 kb) [file 13046_2019_1414_MOESM7_ESM.tif]
