# Supplementary material for: FUBP1 promotes neuroblastoma proliferation via enhancing glycolysis-a new possible marker of malignancy for neuroblastoma
Source: J Exp Clin Cancer Res. 2019 Sep 11;38:400. doi: 10.1186/s13046-019-1414-6 (PMC6737630; doi:10.1186/s13046-019-1414-6)
Supplement: Supplementary file 8 — Table S1. Clinical and biological characteristics in NB tumor samples. (PDF 158 kb) [file 13046_2019_1414_MOESM8_ESM.pdf]

Supplementary materials

Figure legends

Fig.S1 (A)The arrangement of the NB tissue microarray was shown. (B) Immunohistochemistry staining of n-Myc on NB TMA tissue. NB tissue microassay consists of 123 neuroblastoma tumor tissue samples (NB: n=65; GNB: n=31; GN: n=27). (C)Immunohistochemistry staining of FUBP1 on NB TMA tissue. (D)The NB transcriptome data from GEO database was analyzed, and FUBP1 mRNA expression in the NB INSS stage was shown. (E) NB cells were infected with viruses expressing FUBP1 or with siRNA to knock down FUBP1 for 72 hours, followed by western blot analysis. β-Actin served as a loading control. (F)qPCR analysis of plasmid overexpressing FUBP1 or with siRNA to knock down FUBP1 was shown. (G) NB cell SH-EP was infected with siRNAs to knock down FUBP1 for 48 hours, followed by western blot analysis. β-Actin served as a loading control. Images representative of 3 replicates were shown. Data were presented as mean±SEM. $^{**}p < 0.01$.

Fig.S2 (A) SK-N-BE(2) cells were infected with siRNA to knock down FUBP1 for 72 hours, followed by analysis of cell apoptosis via flow cytometry. (B) Statistical analysis of apoptotic cells rate in SK-N-BE(2) cell. (C) SH-EP cells were infected with viruses overexpressing FUBP1 for 72 hours, followed by analysis of cell apoptosis via flow cytometry. (D) Statistical analysis of apoptotic cells rate in SH-EP cell. (E) SK-N-BE(2) cells were infected with siRNA to knock down FUBP1 for 72 hours, then incubated with hypoxia, followed by analysis of cell apoptosis via flow cytometry. (F) Statistical analysis of apoptotic cells rate in SK-N-BE(2) cell. Samples were subjected to FITC-Annexin V/propidium iodide staining, and the quantitative analysis of apoptotic cells was performed using flow cytometry. The dotted plots are divided into double negative (live cells), Annexin V negative PI positive (necrotic cells), Annexin V positive PI negative (early apoptosis cells) and double positive (late apoptosis cells). Diagrams of FITC-Annexin V/propidium iodide flow cytometry in a representative experiment are presented as the above graphs.

Fig.S3 (A)Immunohistochemistry staining of LDHA on NB TMA tissue. (B)Statistical analysis of histochemistry score (H-Score) of LDHA in TMA. (C) Survival of NB patients (including 3GN, 23GNB, 44NB) with high expressed LDHA (H-Score ≥5) versus those with low expressed LDHA (H-Score<5). (D) Survival of NB patients (Pathological pattern is NB not GN or GNB) with high expressed LDHA (H-Score ≥5) versus those with low expressed LDHA (H-Score<5). (E) Correlation analysis of LDHA and FUBP1 expression in NB TMA tissue. Data were presented as mean±SEM. $^{*}p< 0.05$.

Fig.S4 (A) Immunohistochemistry staining of c-Myc on NB TMA tissue. (B)Statistical analysis of histochemistry score (H-Score) of c-Myc in TMA. (C) Correlation analysis of c-Myc and FUBP1 expression in NB TMA tissue. (D) SH-EP cell was infected with siRNAs to knock down c-Myc, HIF1α and both were knocked down simultaneously for 24h, then transfected with overexpressing FUBP1 plasmid for 24h. β-Actin served as a loading control. (E) NB cell SH-EP was infected with siRNAs to knock down HIF1α or c-Myc for 48 hours, followed by western blot analysis. β-Actin served as a loading control. Images representative of 3 replicates were shown. Data were presented as mean±SEM. $^{*}p< 0.05$.

Fig.S5 (A) Statistical analysis of histochemistry score (H-Score) of n-Myc in TMA. (B) SH-EP cell was infected with viruses over-expressing n-Myc or with siRNA to knock down n-Myc for 48 hours, followed by western blot analysis to detect FUBP1, c-Myc and LDHB levels. (C) The interfering effect of siRNAs to knock down n-Myc was detected by western blot analysis in the SH-EP cell. β-Actin served as a loading control. (D) SH-EP cell was infected with siRNAs to knock down FUBP1 and plasmid to overexpress FUBP1 for 24h, then n-Myc levels were detected by western blot. β-Actin served as a loading control. Images representative of 3 replicates were shown. Data were presented as mean±SEM. $^{**}p< 0.01$.

Fig.S6 (A) qPCR analysis of Hif1α gene expression in the SH-EP cell, followed by transfecting with viruses expressing FUBP1 or with siRNA to knock down FUBP1 was shown. (B) 293T cell was infected with FUBP1 overexpressing plasmid or empty vector plasmid, Hif1α promoter luciferase reporter plasmid and renilla luciferase

plasmid for 48h, followed by fluorescence detection. Renilla luciferase served as the transfection control. Images representative of 3 replicates were shown. Data were presented as mean±SEM. $^{*}p < 0.05$, $^{**}p < 0.01$.

Fig.S7 (A) qPCR analysis of Vhl gene expression in the SH-EP cell, followed by transfecting with viruses expressing FUBP1 or with siRNA to knock down FUBP1 was shown. (B) Chip assays were performed to verify whether FUBP1 bound to Vhl promoter other sequences (-1434bp ~ -1326bp and -545bp ~ -433bp). Images representative of 3 replicates were shown. Data were presented as mean ± SEM. $^{*}p < 0.05$, $^{***}p < 0.001$.

Table S1 Clinical and biological characteristics in NB tumor samples

Clinical diagnosis	Age	Gender	n-myc status	INSS	Total number
NB	<547 days 46	Female 46	Amplified 8	I−III 50	**65**
	≥547 days 19	Male 19	Non-amplified 37	IV 15	
GNB	<547 days 6	Female 16	Amplified 2	I-III 24	**31**
	≥547 days 25	Male 15	Non-amplified 17	IV 7	
GN	<547 days −	Female 13	Amplified −	I-III 21	**27**
	≥547 days 27	Male 14	Non-amplified 3	IV −	
